# Supplementary material for: Comparing Zwitterionic and PEG Exteriors of Polyelectrolyte Complex Micelles
Source: Molecules. 2020 May 30;25(11):2553. doi: 10.3390/molecules25112553 (PMC7321349; doi:10.3390/molecules25112553)
Supplement: Supplementary file 1 [file molecules-25-02553-s001.zip › molecules-812712-supplementary-final.pdf]

molecules

Supporting Information

Comparing Zwitterionic and PEG Exteriors of Polyelectrolyte Complex Micelles

Jeffrey M. Ting [1,2,†], Alexander E. Marras [1,2,†], Joseph D. Mitchell [1], Trinity R. Campagna [1] and Matthew V. Tirrell [1,2,*]

[1] Pritzker School of Molecular Engineering, University of Chicago, Chicago, IL 60637, USA, jting1@uchicago.edu (J.M.T.); marras@uchicago.edu (A.E.M.); jdm41297@gmail.com (J.D.M.); trinityc@uchicago.edu (T.R.C.); mtirrell@uchicago.edu (M.V.T.)

[2] Center for Molecular Engineering and Materials Science Division, Argonne National Laboratory, Lemont, IL 60439, USA.

† These authors contributed equally to this work.

Contents

S1. Supplemental Polymer Synthesis Data (Figures S1 to S8)
S2. Supplemental Dynamic Light Scattering Data (Figures S9 to S39)
S3. Supplemental Small-Angle X-Ray Scattering Data (Figures S40 to S46, Table S1)
S4. Supplemental Polyelectrolyte Complex Micelle Stability Data (Figure S47)

S1. Supplemental Polymer Synthesis Data

PMPC-PVBTMA Synthesis. Figure S-1 shows a representative crude ^{1}H NMR of PMPC, resulting in 81% total monomer conversion after 18 h. Figure S-2 shows a representative crude ^{1}H NMR of PMPC-PVBTMA, resulting in 93% total monomer conversion after 18 h. Figure S-3 shows the purified ^{1}H NMR of PMPC$_{5K}$-PVBTMA$_{50}$.

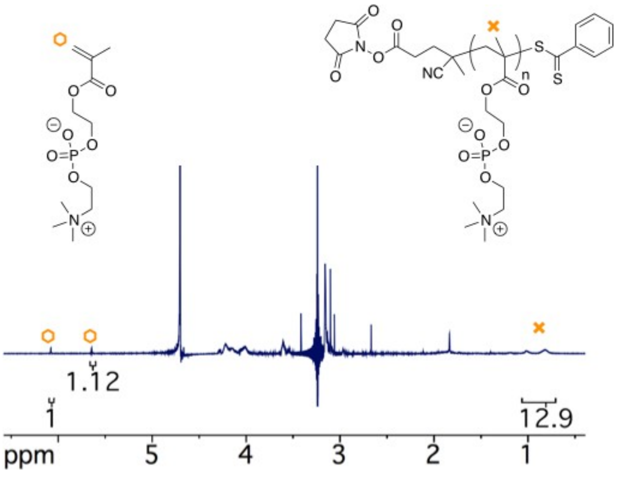

Figure S1. Crude ^{1}H NMR of PMPC in D_2O.

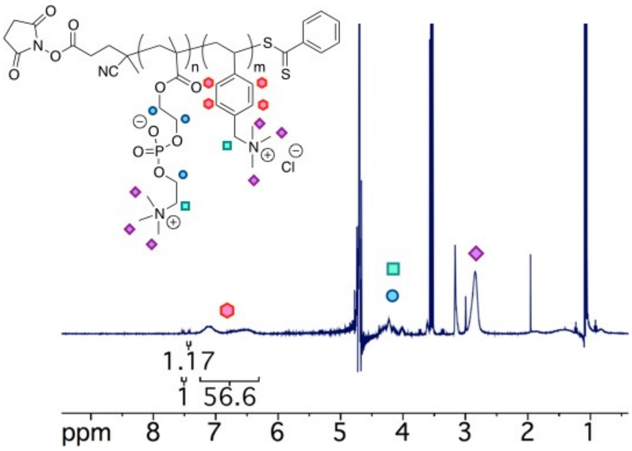

Figure S2. Crude ^{1}H NMR of PMPC-PVBTMA in D_2O.

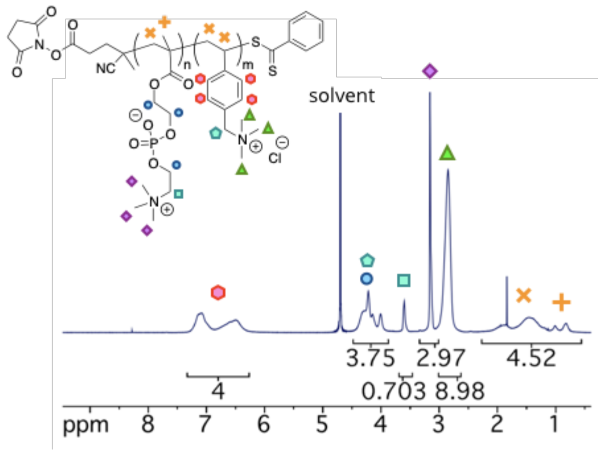

Figure S3. ^{1}H NMR of $PMPC_{5K}$-$PVBTMA_{50}$ in D_2O.

PAA Synthesis. Figures S-4 and S-5 show the ^{1}H NMR of the BuPA RAFT CTA and the homopolymer PAA prepared with aqueous RAFT polymerization, respectively. End-group analysis (51 × 94.04 g/mol + 238.39 g/mol) resulted in a calculated M_n = 5030 g/mol, in excellent agreement with SEC-MALS characterization. Figure S-6 shows the SEC refractive index trace of PAA, which consists of a monomodal peak exhibiting narrow dispersity in the M_n distribution.

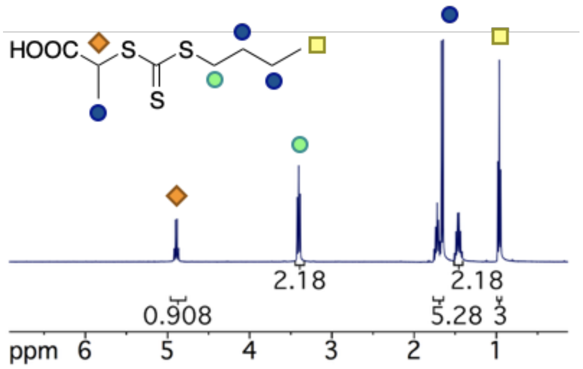

Figure S4. ^{1}H NMR of BuPA in $CDCl_3$.

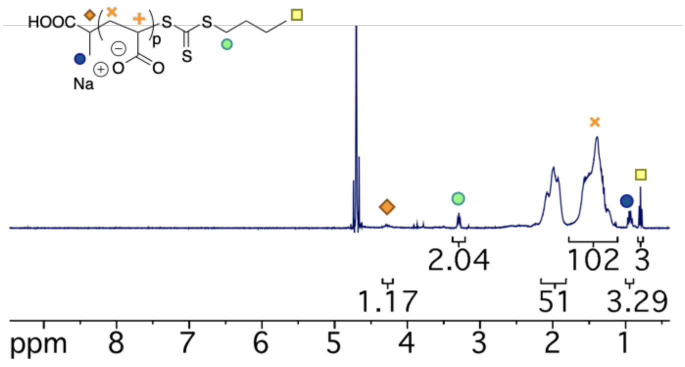

Figure S5. ^1H NMR of PAA in D_2O.

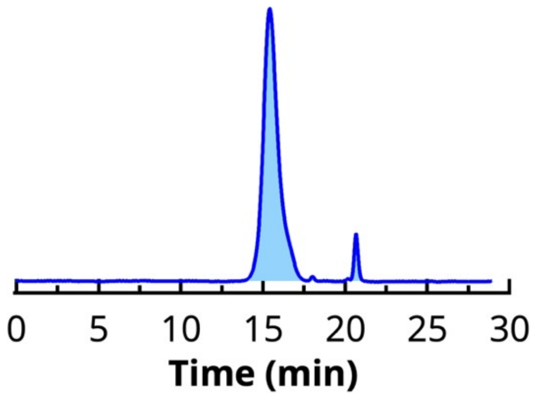

Figure S6. SEC refractive index trace of PAA (M_n = 4900 g/mol, $Đ$ = 1.11).

Refractive Index Measurements. To determine the absolute M_n of the polymers measured by SEC-MALS, we employed a refractometer to measure the dn/dc of polymers in their respective mobile phase. For PMPC-PVBTMA samples, Figure S-7 shows the dn/dc determination of the individual homopolymers. Using Equation 1 shown in the main manuscript, we calculated the dn/dc of the $PMPC_{5K}$-$PVBTMA_{50}$ and $PMPC_{10K}$-$PVBTMA_{100}$ to be 0.1661 and 0.1663 mL/g, respectively.

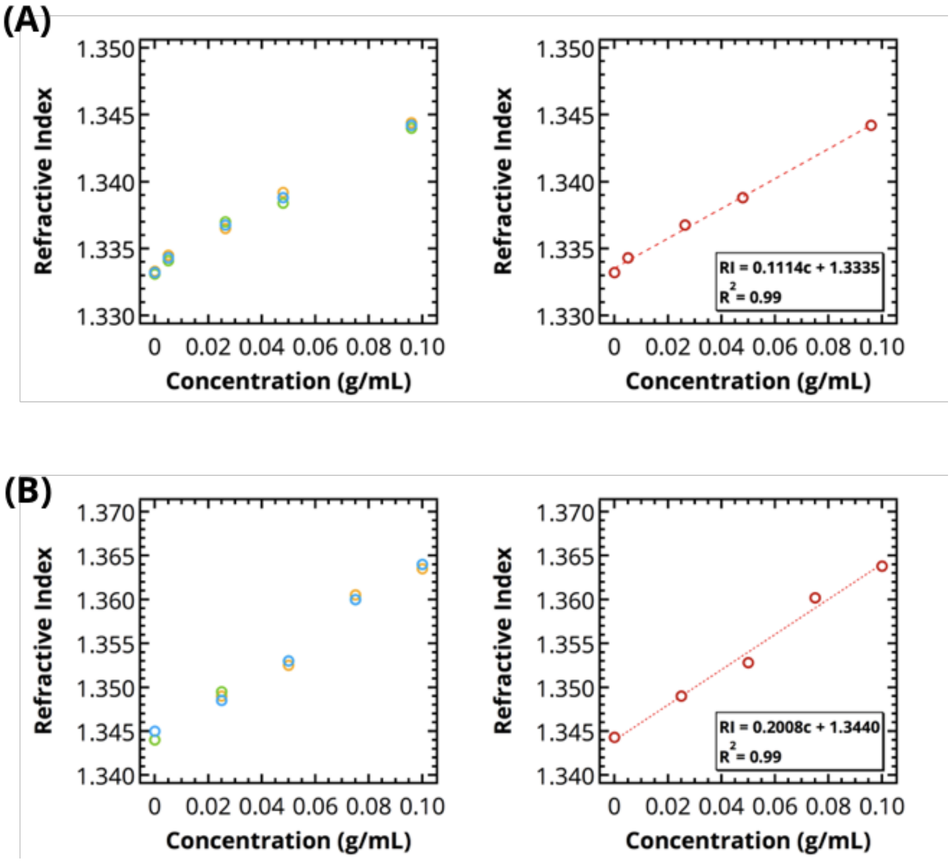

Figure S7. Measured refractive index values versus polymer concentration of (A) PMPC and (B) PVBTMA. All measurements were taken using polymers completely dissolved in the cationic mobile phase solution at 25 °C. The circles on the left plot show the raw data; the circles on the right plot show the average of triplicate measurements with the dashed line denoting a linear regression to determine d*n*/dc.

Thermogravimetric Analysis Curves. Figure S-8 shows the TGA profiles of synthesized polyelectrolytes. All experiments were conducted at 15 °C/min.

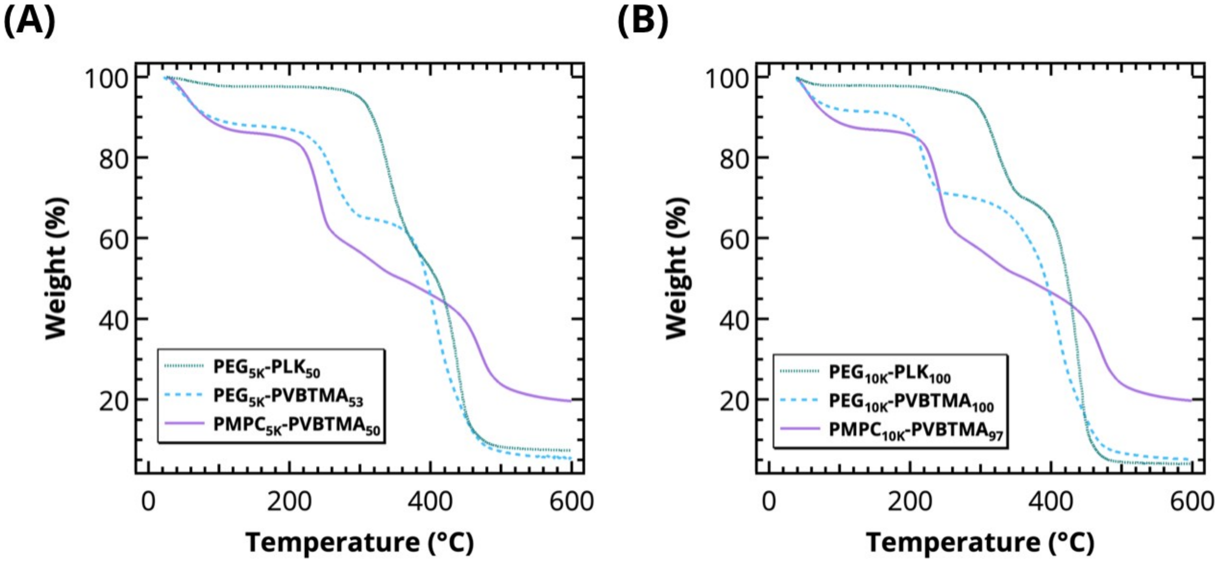

Figure S8. TGA curves for block polymers (A) PEG_{5K}-PLK_{50}, PEG_{5K}-$PVBTMA_{53}$ and $PMPC_{5K}$-$PVBTMA_{50}$, as well as (B) PEG_{10K}-PLK_{100}, PEG_{10K}-$PVBTMA_{100}$ and $PMPC_{10K}$-$PVBTMA_{97}$. All experiments were conducted at 15 °C/min.

S2. Supplemental Dynamic Light Scattering Data

Figure S-9 shows a gallery of histograms for all prepared PCMs from 0-mM NaCl to 200-mM NaCl. The autocorrelation functions for these samples are shown in Figures S35 through S38 below.

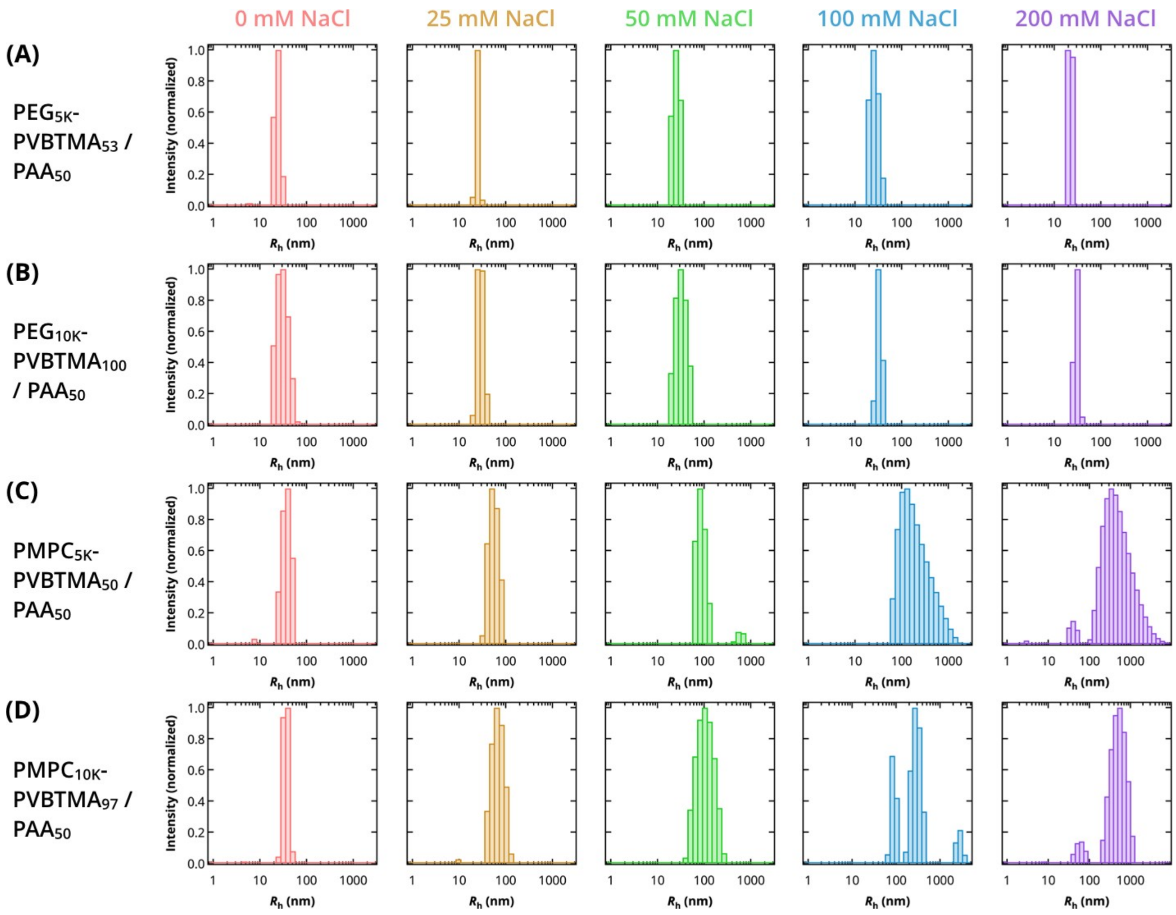

Figure S9. Apparent size hydrodynamic radius distribution of (**A**) PEG_{5K}-$PVBTMA_{53}$ / PAA_{50},(**B**) PEG_{10K}-$PVBTMA_{100}$ / PAA_{50}, (**C**) $PMPC_{5K}$-$PVBTMA_{50}$ / PAA_{50} and (**D**) $PMPC_{10K}$- $PVBTMA_{97}$ / PAA_{50} as NaCl salt is increased from 0 mM to 200-mM (left to right).

The detailed multi-angle DLS analysis of the PCMs is provided below. For each polymer system, Figures S10 through S33 show the measured angular dependence of the autocorrelation functions between 60° and 120° fitted by a cumulant expansion, as well as the linear regression of Γ vs q^2 between 60° and 120°.

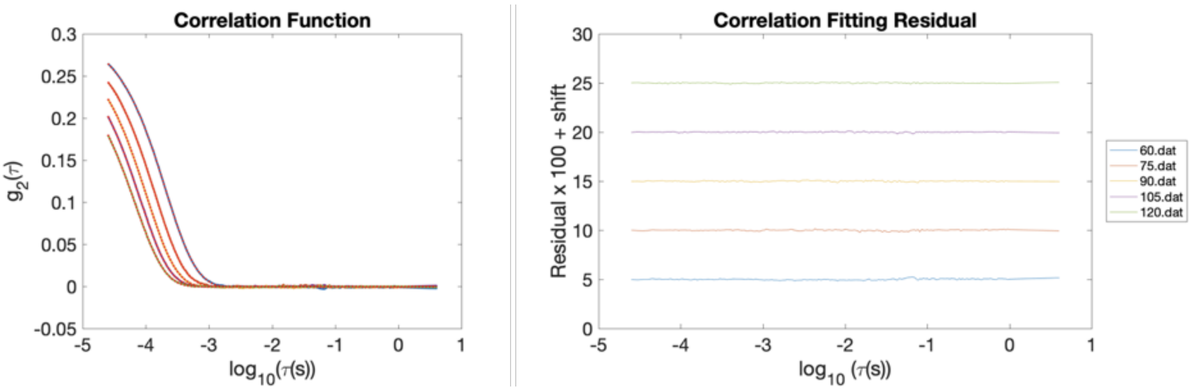

Figure S10. Measured angular dependence of the autocorrelation function for PEG_{5K}-PLK_{47} / PAA_{50} at 0-mM NaCl between 60° and 120° fitted by cumulant expansion.

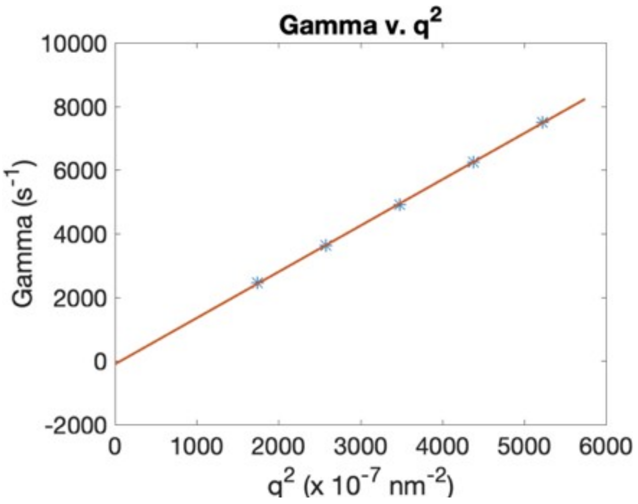

Figure S11. Linear regression of Γ vs q^2 for PEG_{5K}-PLK_{47} / PAA_{50} at 0-mM NaCl over 5 angles between 60° and 120°.

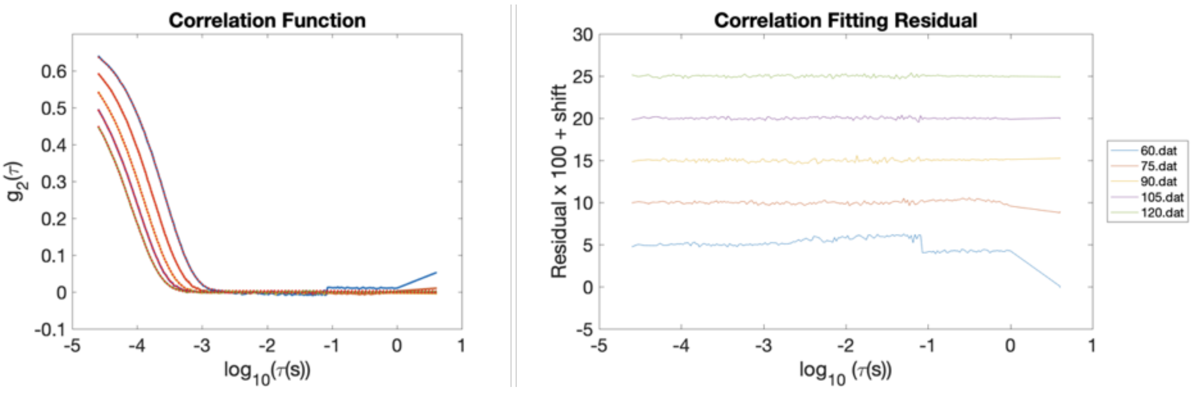

Figure S12. Measured angular dependence of the autocorrelation function for PEG_{5K}-PLK_{47} / PAA_{50} at 100-mM NaCl between 60° and 120° fitted by cumulant expansion.

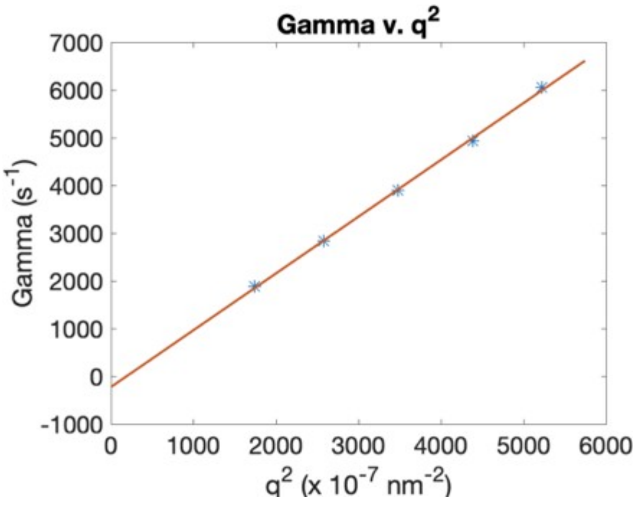

Figure S13. Linear regression of Γ vs q^2 for PEG_{5K}-PLK_{47} / PAA_{50} at 100-mM NaCl over 5 angles between 60° and 120°.

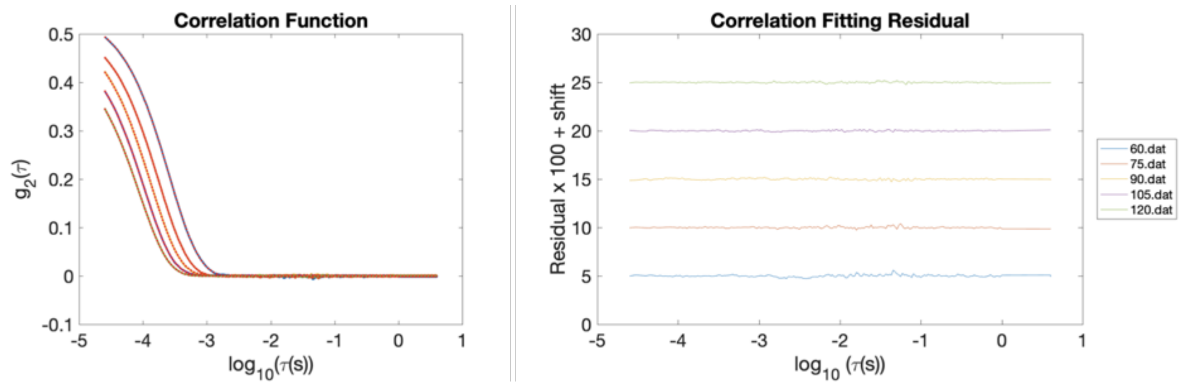

Figure S14. Measured angular dependence of the autocorrelation function for PEG_{10K}-PLK_{93} / PAA_{50} at 0-mM NaCl between 60° and 120° fitted by cumulant expansion.

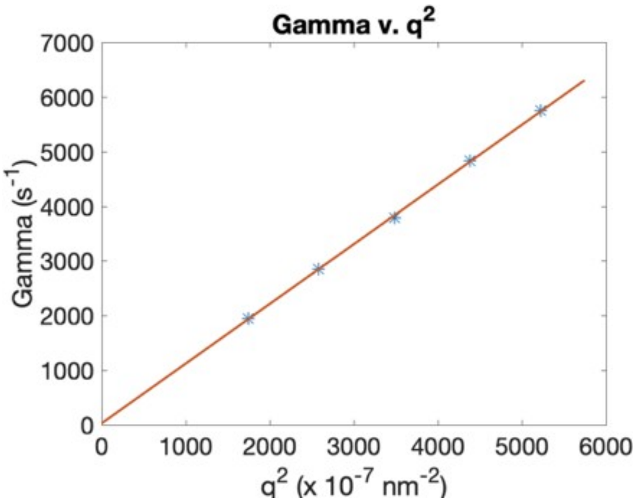

Figure S15. Linear regression of Γ vs q^2 for PEG_{10K}-PLK_{93} / PAA_{50} at 0-mM NaCl over 5 angles between 60° and 120°.

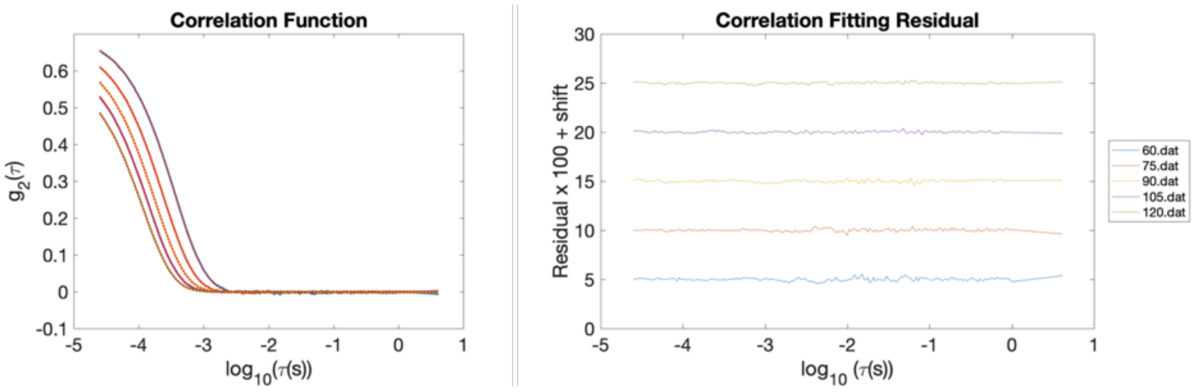

Figure S16. Measured angular dependence of the autocorrelation function for PEG_{10K}-PLK_{93} / PAA_{50} at 100-mM NaCl between 60° and 120° fitted by cumulant expansion.

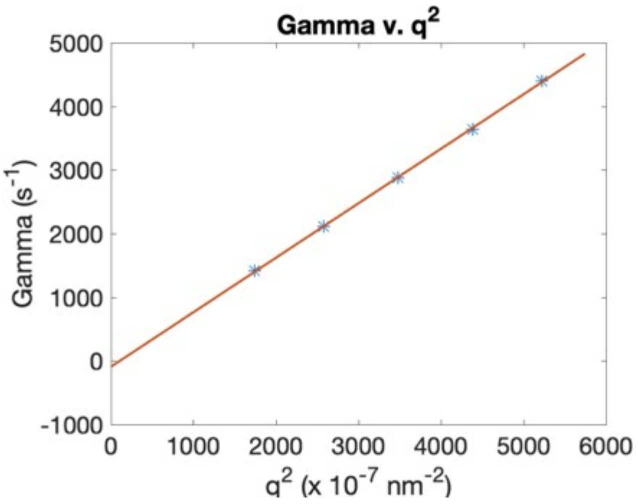

Figure S17. Linear regression of Γ vs q^2 for PEG_{10K}-PLK_{93} / PAA_{50} at 100-mM NaCl over 5 angles between 60° and 120°.

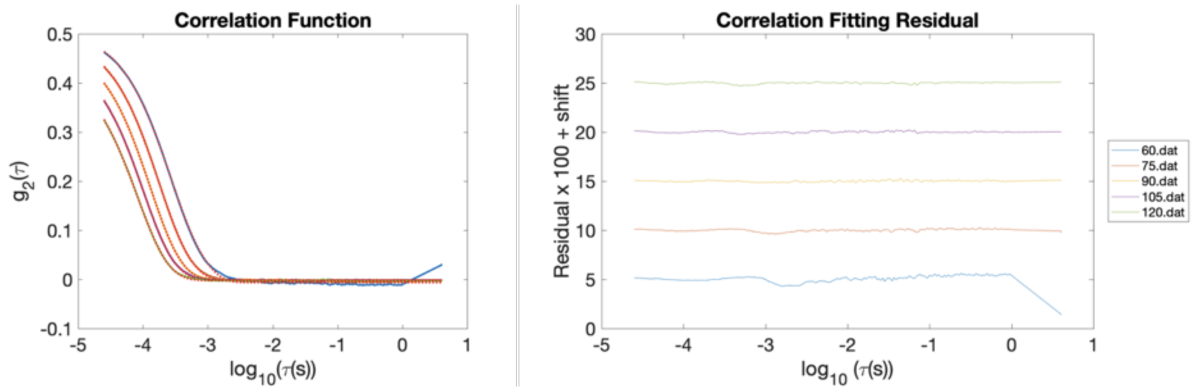

Figure S18. Measured angular dependence of the autocorrelation function for PEG_{5K}-$PVBTMA_{53}$/ PAA_{50} at 0-mM NaCl between 60° and 120° fitted by cumulant expansion.

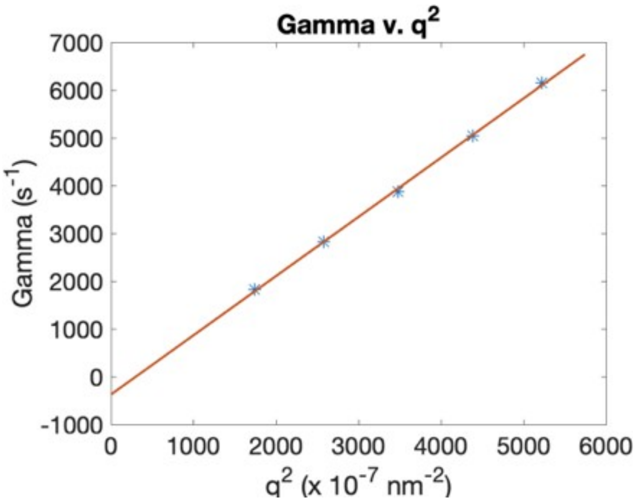

Figure S19. Linear regression of Γ vs q^2 for PEG_{5K}-$PVBTMA_{53}$ / PAA_{50} at 0-mM NaCl over 5 angles between 60° and 120°.

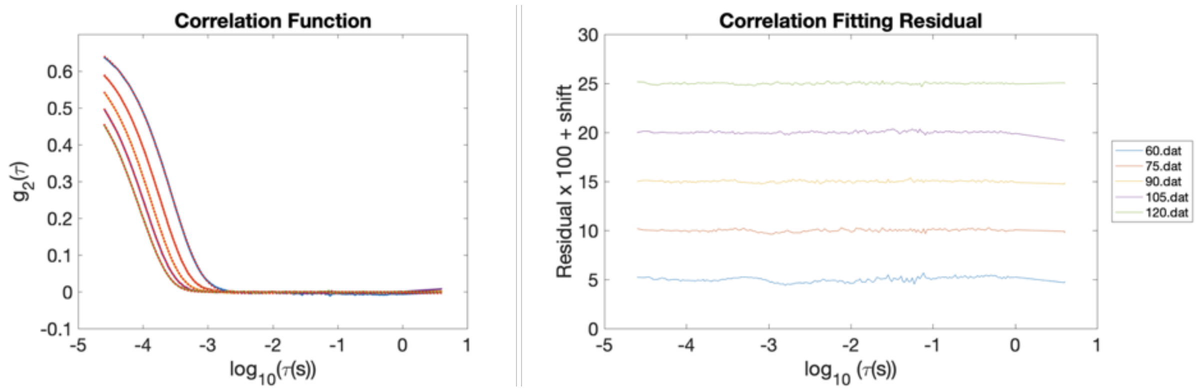

Figure S20. Measured angular dependence of the autocorrelation function for PEG$_{5K}$-PVBTMA$_{53}$/ PAA$_{50}$ at 100-mM NaCl between 60° and 120° fitted by cumulant expansion.

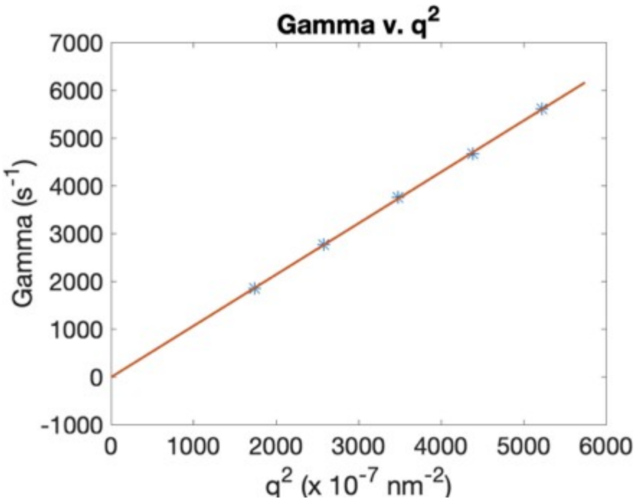

Figure S21. Linear regression of Γ vs q^2 for PEG$_{5K}$-PVBTMA$_{53}$ at 100-mM NaCl over 5 angles between 60° and 120°.

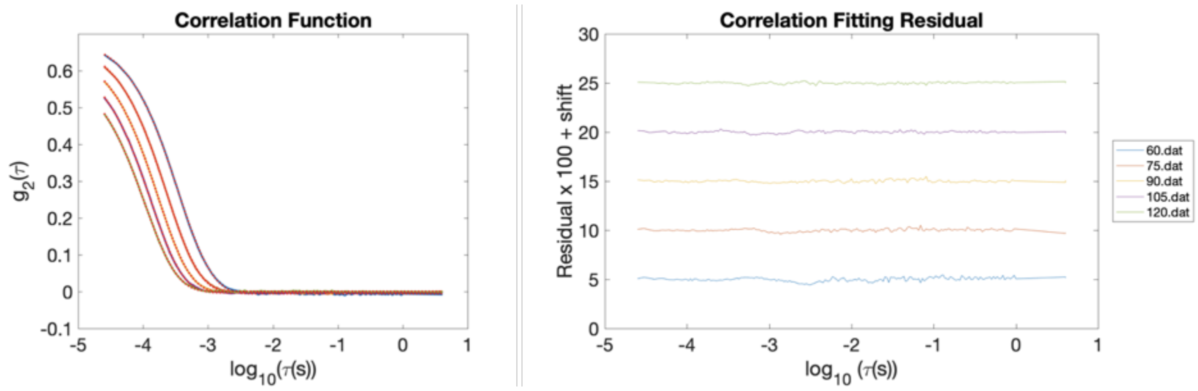

Figure S22. Measured angular dependence of the autocorrelation function for PEG$_{10K}$- PVBTMA$_{100}$ / PAA$_{50}$ at 0-mM NaCl between 60° and 120° fitted by cumulant expansion.

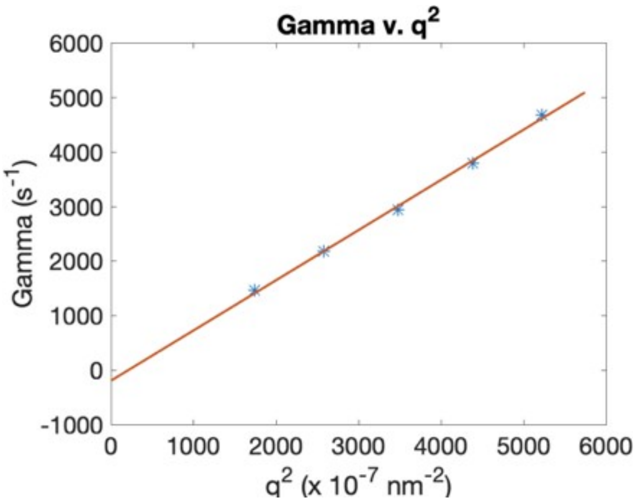

Figure S23. Linear regression of Γ vs q^2 for PEG_{10K}-$PVBTMA_{100}$ / PAA_{50} at 0-mM NaCl over 5 angles between 60° and 120°.

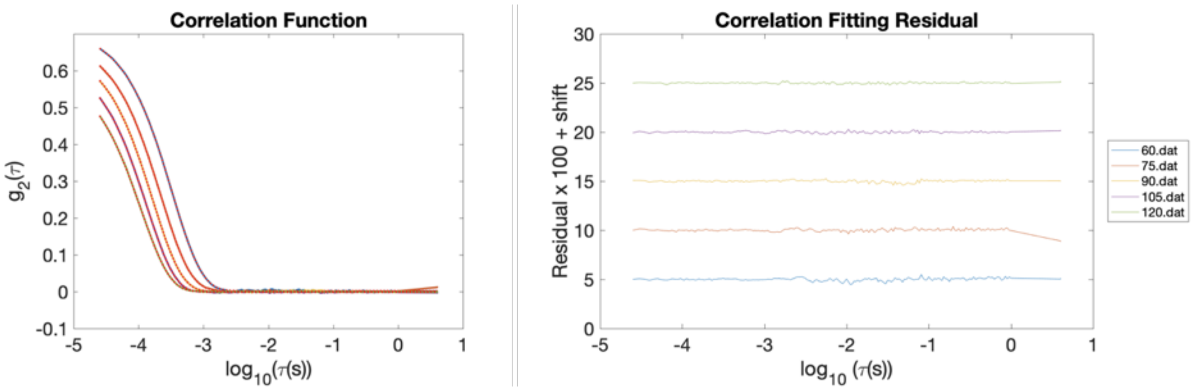

Figure S24. Measured angular dependence of the autocorrelation function for PEG_{10K}- $PVBTMA_{100}$ / PAA_{50} at 100-mM NaCl between 60° and 120° fitted by cumulant expansion.

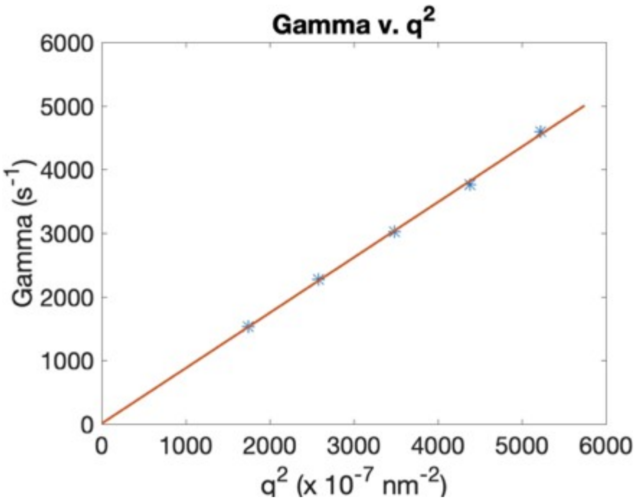

Figure S25. Linear regression of Γ vs q^2 for PEG_{10K}-$PVBTMA_{100}$ / PAA_{50} at 100-mM NaCl over 5 angles between 60° and 120°.

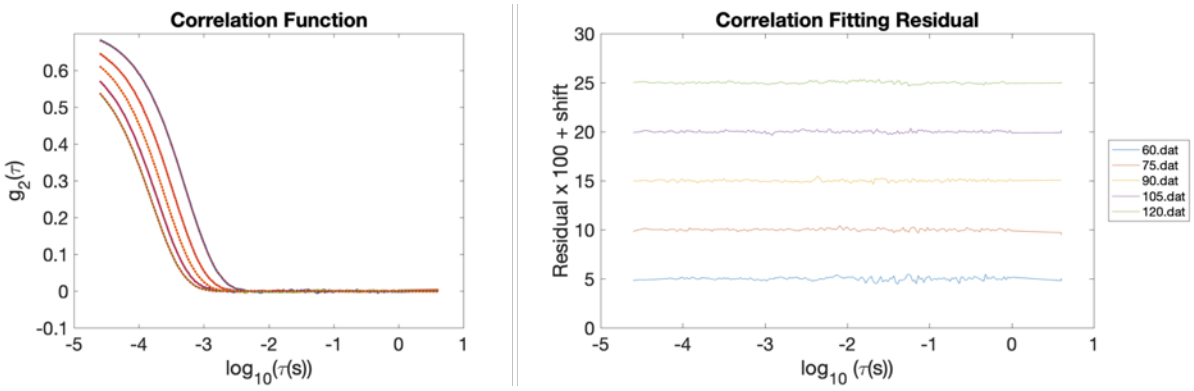

Figure S26. Measured angular dependence of the autocorrelation function for $PMPC_{5K}$-$PVBTMA_{50}$ / PAA_{50} at 0-mM NaCl between 60° and 120° fitted by cumulant expansion.

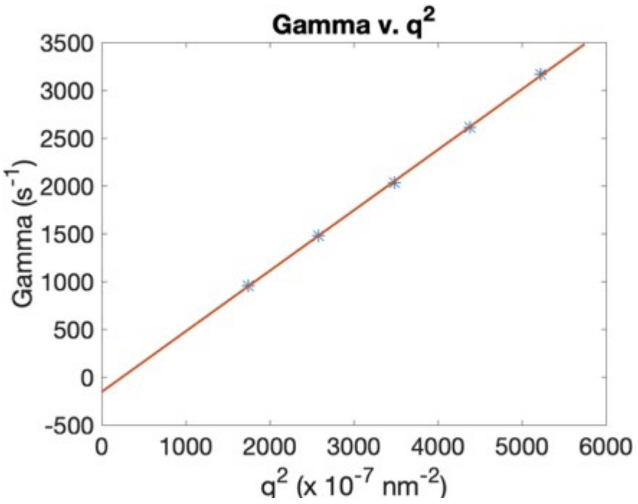

Figure S27. Linear regression of Γ vs q^2 for $PMPC_{5K}$-$PVBTMA_{50}$ / PAA_{50} at 0-mM NaCl over 5 angles between 60° and 120°.

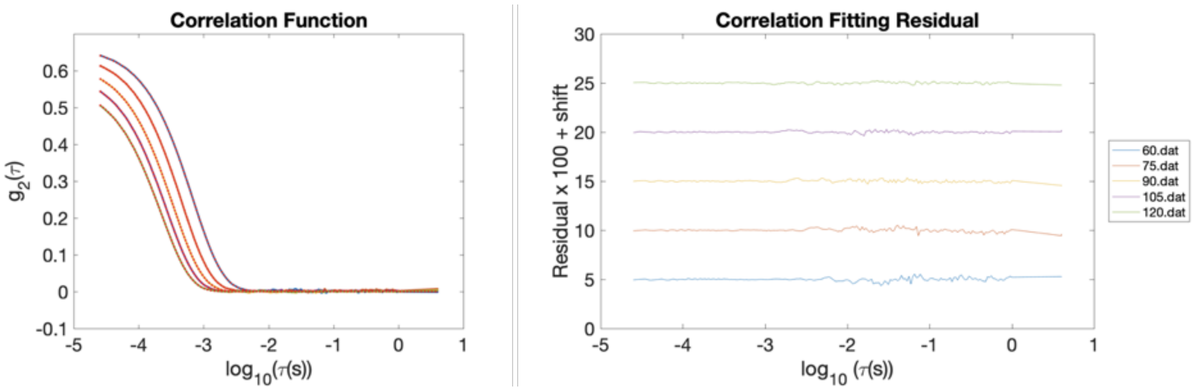

Figure S28. Measured angular dependence of the autocorrelation function for $PMPC_{5K}$-$PVBTMA_{50}$ / PAA_{50} at 100-mM NaCl between 60° and 120° fitted by cumulant expansion.

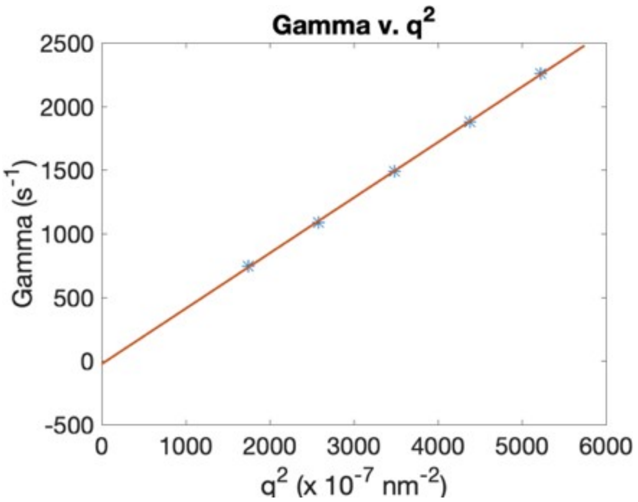

Figure S29. Linear regression of Γ vs q^2 for $PMPC_{5K}$-$PVBTMA_{50}$ / PAA_{50} at 100-mM NaCl over 5 angles between 60° and 120°.

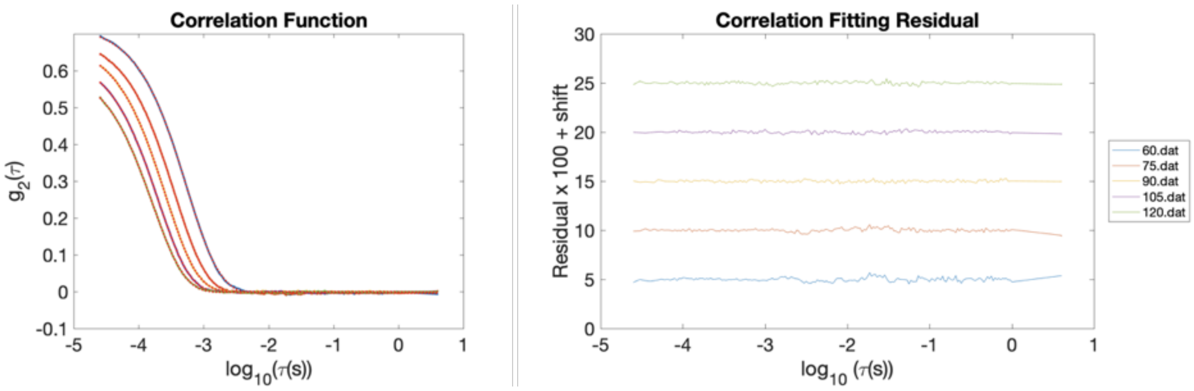

Figure S30. Measured angular dependence of the autocorrelation function for $PMPC_{10K}$- $PVBTMA_{97}$ / PAA_{50} at 0-mM NaCl between 60° and 120° fitted by cumulant expansion.

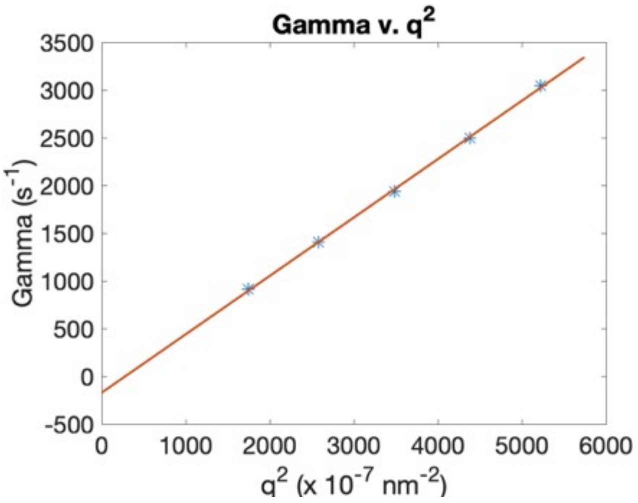

Figure S31. Linear regression of Γ vs q^2 for $PMPC_{10K}$-$PVBTMA_{97}$ / PAA_{50} at 0-mM NaCl over 5 angles between 60° and 120°.

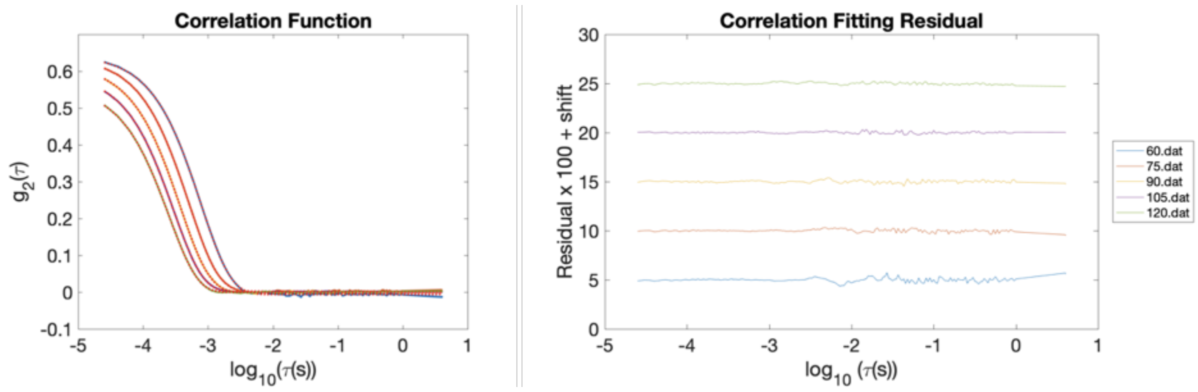

Figure S32. Measured angular dependence of the autocorrelation function for PMPC$_{10K}$-PVBTMA$_{97}$ / PAA$_{50}$ at 100-mM NaCl between 60° and 120° fitted by cumulant expansion.

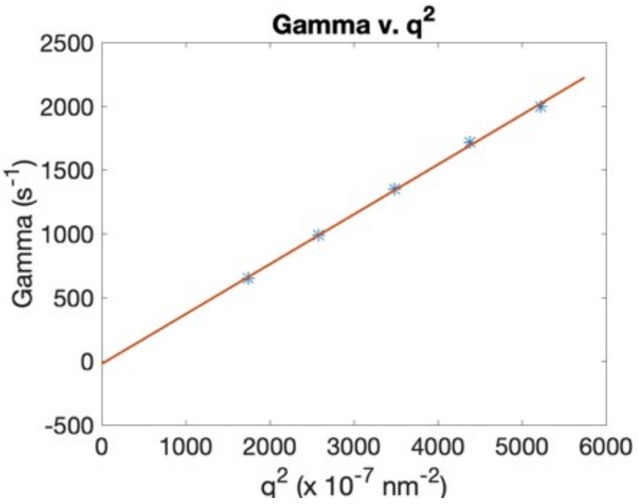

Figure S33. Linear regression of Γ vs q^2 for PMPC$_{10K}$-PVBTMA$_{97}$ / PAA$_{50}$ at 100-mM NaCl over 5 angles between 60° and 120°.

Figures S-34 through S-37 show the autocorrelation functions that correspond to Figure S-9 for all investigated micelle systems from 0 to 200-mM NaCl.

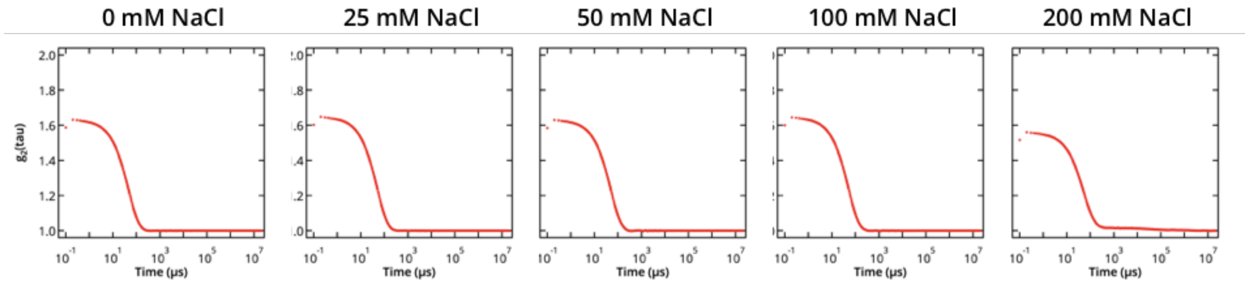

Figure 34. Summary of the autocorrelation functions of PCM assemblies PEG$_{5K}$-PVBTMA$_{53}$ at 0-200 mM NaCl.

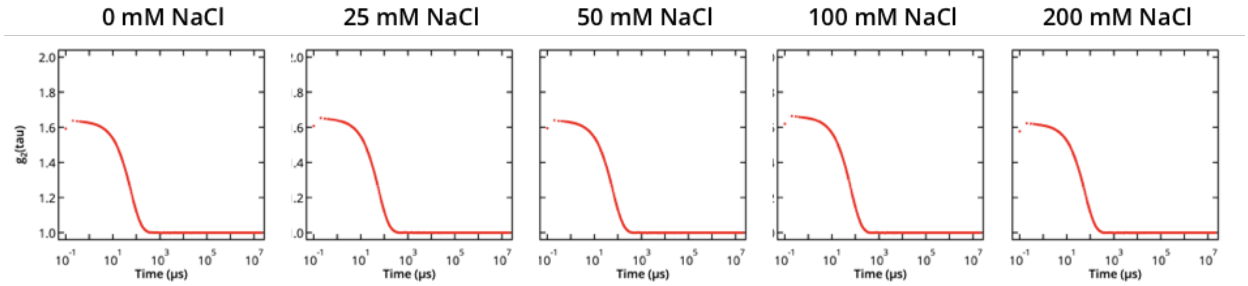

Figure S35. Summary of the autocorrelation functions of PCM assemblies PEG$_{10K}$-PVBTMA$_{100}$ at 0-200 mM NaCl.

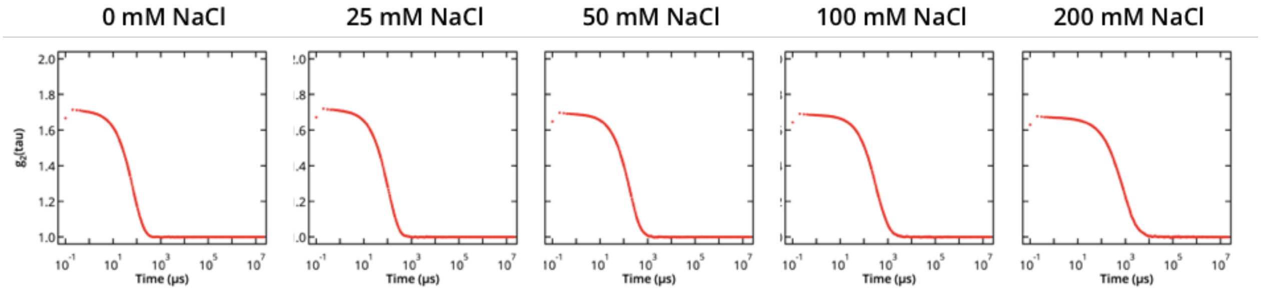

Figure S36. Summary of the autocorrelation functions of PCM assemblies PMPC$_{5K}$-PVBTMA$_{50}$ at 0-200 mM NaCl.

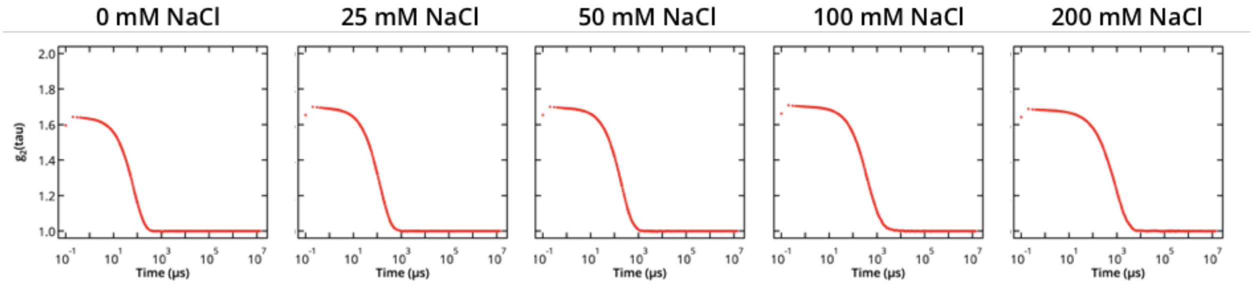

Figure S37. Summary of the autocorrelation functions of PCM assemblies PMPC$_{10K}$-PVBTMA$_{97}$ at 0-200 mM NaCl.

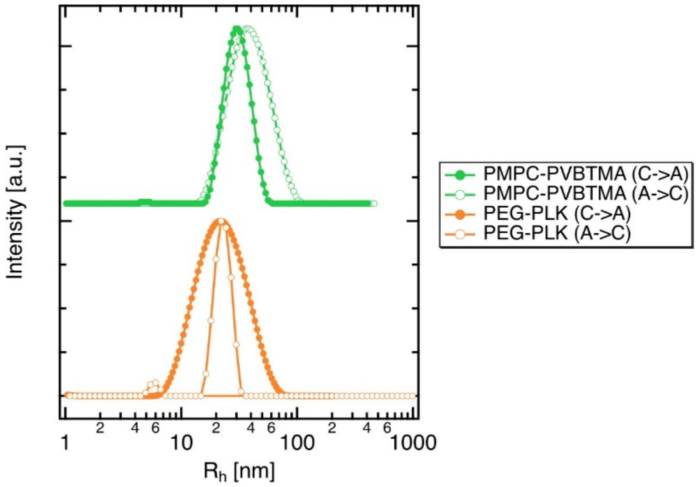

Figure S38. REPES fits for DLS of PCMs assembled in different orders. All micelles in this work were assembled with the cation added to the solution first, followed by the anion (C→A). When this order is reversed (A→C) the size distribution is different, suggesting that the system may be kinetically trapped.

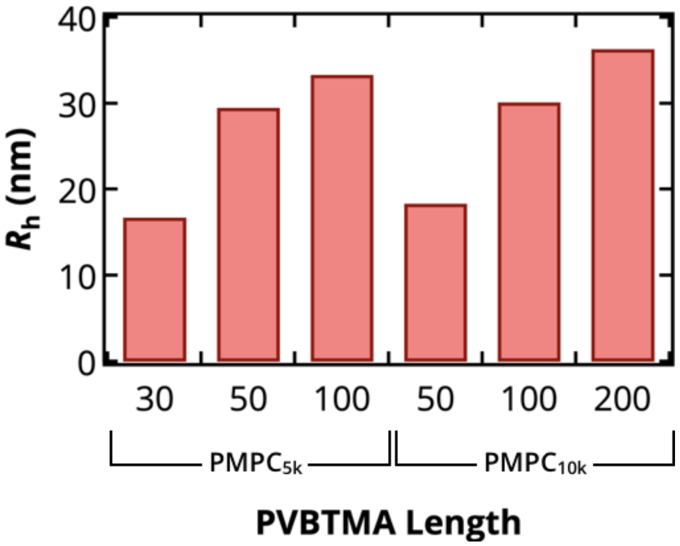

Figure S39. Comparison of the apparent hydrodynamic radius versus charged block length of PVBTMA in PCM assemblies.

S3. Supplemental Small-Angle X-ray Scattering

Figure S40 shows the intensity versus q profiles of the PEG-PLK, PEG-PVBTMA and PMPC-PVBTMA polyelectrolyte solutions with increasing NaCl salt. At 0-mM NaCl, the correlation peak can be observed at q = 0.4–0.5 Å for PEG-PLK and q = 0.2–0.4 Å for PEG-PVBTMA and PMPC-PVBTMA. Table S-2 contains the fitting for the SAXS data in Figure 7 of the main manuscript with added $MgCl_2$ salt.

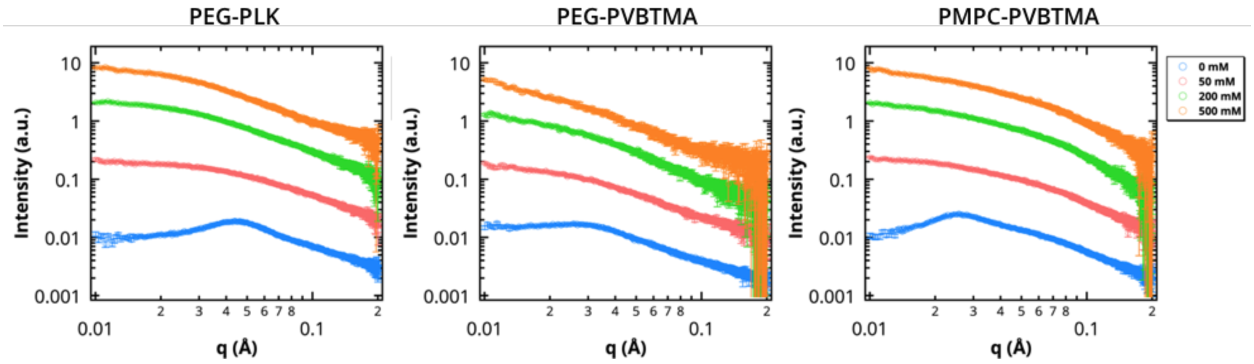

Figure S40. SAXS profiles of polyelectrolyte solutions of PEG-PLK, PEG-PVBTMA and PMPC-PVBTMA at polymer concentrations of 5 mg/mL with increasing added NaCl salt. Intensity scaled for clarity.

Table S1. SAXS summary with added $MgCl_2$ salt.

Sample	$[MgCl_2]$ (mM)	*R*Guinier [a] (nm)	PDI [b]
PEG_{5K}-PLK_{47} / PAA_{50}	50	8.0	0.16
	100	8.6	0.14
PEG_{10K}-PLK_{93} / PAA_{50}	50	11.2	0.15
	100	10.6	0.09
PEG_{5K}-$PVBTMA_{53}$ / PAA_{50}	50	10.7	0.09
	100	-	-
PEG_{10K}-$PVBTMA_{100}$ / PAA_{50}	50	19.8	0.15
	100	-	-
$PMPC_{5K}$-$PVBTMA_{50}$ / PAA_{50}	50	-	-
	100	-	-
$PMPC_{10K}$-$PVBTMA_{97}$ / PAA_{50}	50	-	-
	100	-	-

[a] Mean radius from Guinier fit; predominately core (nm) [b] Polydispersity index (σ^2/R^2). Models use Schulz-Zimm distribution flexible cylinder and Unified Level fits, except for PEG_{10K}-$PVBTMA_{47}$ / PAA_{100} (spheroid) and PEG_{10K}-PLK_{93} / PAA_{50} (cylinder fitting).

For each polymer system, Figures S-41 through S-46 show the small angle X-ray scattering profile in water with 100, 250 and 500 mM NaCl as well as 50 and 100 mM $MgCl_2$.

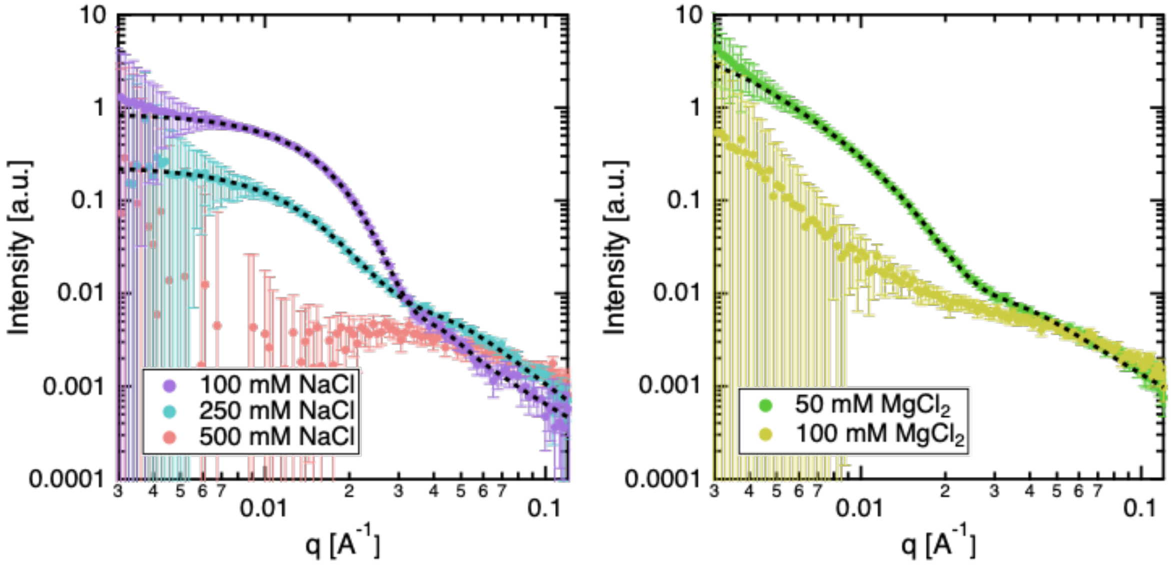

Figure S41. SAXS profiles for PEG_{5K}-$PVBTMA_{53}$ / PAA_{50} with 100, 250 and 500-mM NaCl (left), as well as 50 and 100-mM $MgCl_2$ (right).

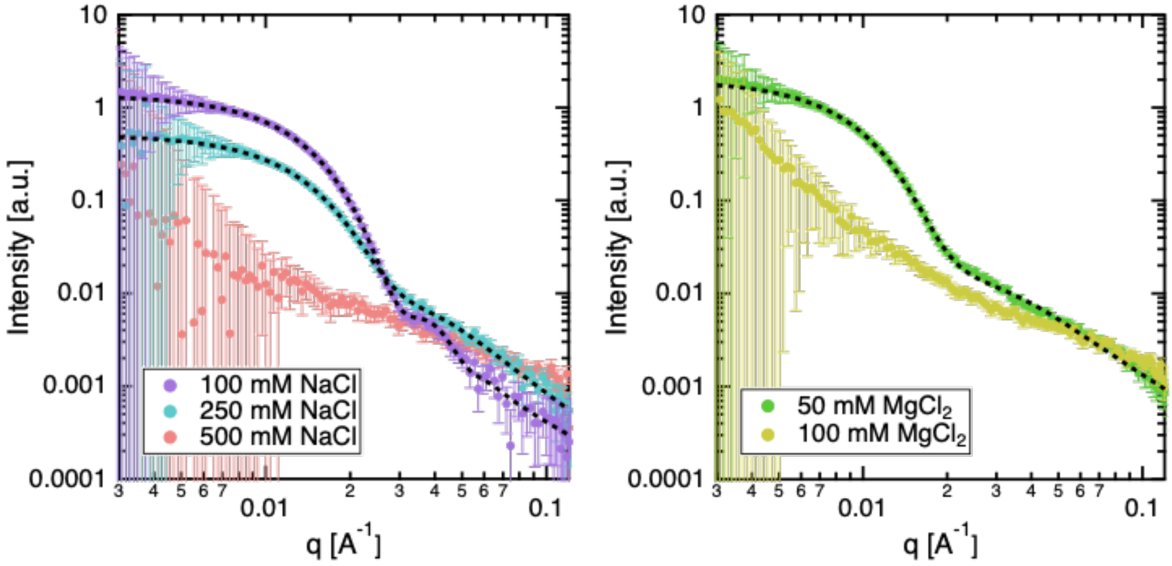

Figure S42. SAXS profiles for PEG_{10K}-$PVBTMA_{100}$ / PAA_{50} with 100, 250 and 500-mM NaCl (left), as well as 50 and 100-mM $MgCl_2$ (right).

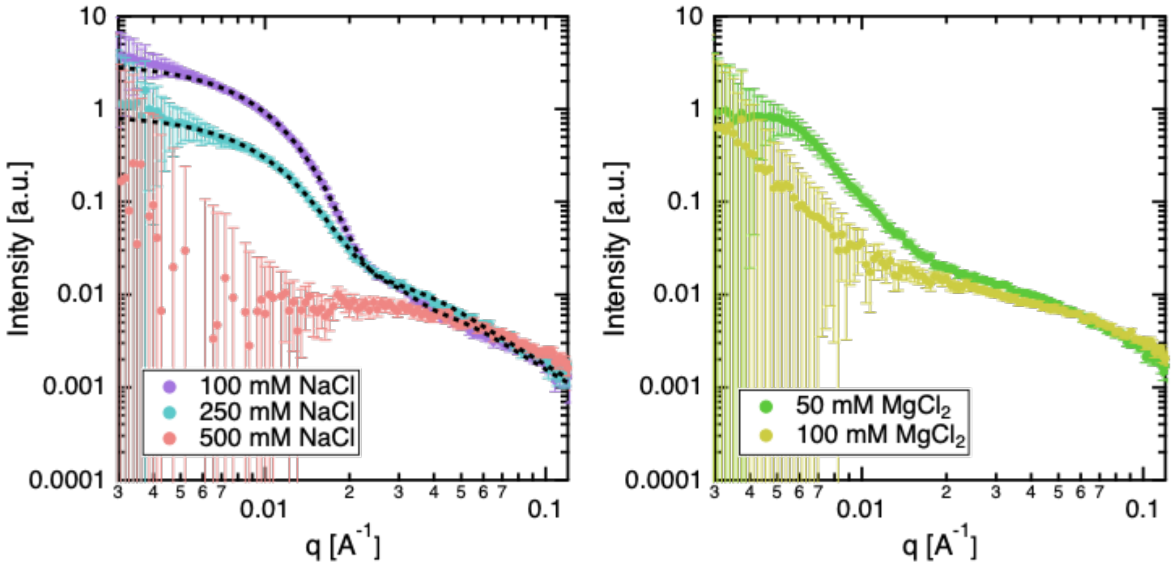

Figure S43. SAXS profiles for PMPC$_{5K}$-PVBTMA$_{50}$ / PAA$_{50}$ with 100, 250 and 500-mM NaCl (left), as well as 50 and 100-mM $MgCl_2$ (right).

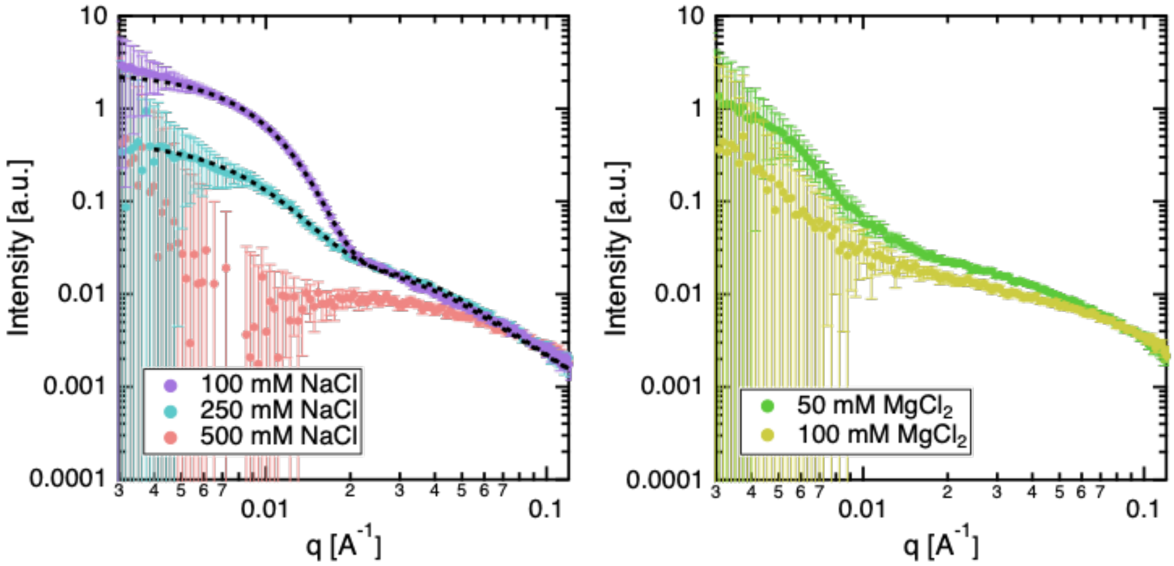

Figure S44. SAXS profiles for PMPC$_{10K}$-PVBTMA$_{97}$ / PAA$_{50}$ with 100, 250 and 500-mM NaCl (left), as well as 50 and 100-mM $MgCl_2$ (right).

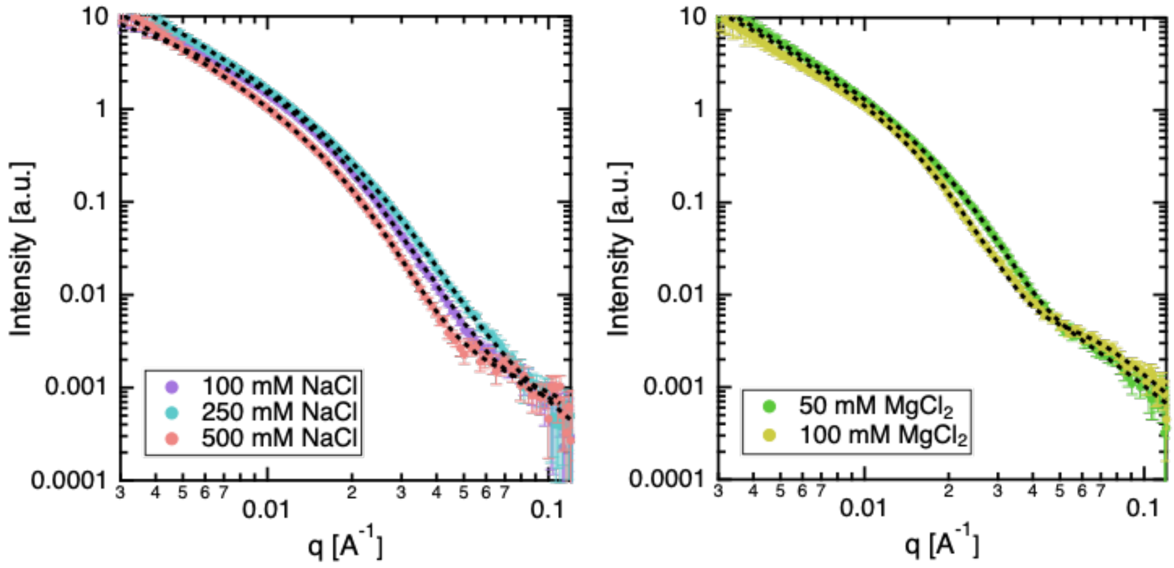

Figure S45. SAXS profiles for PEG_{5K}-PLK_{47} / PAA_{50} with 100, 250 and 500-mM NaCl (left), as well as 50 and 100-mM $MgCl_2$ (right).

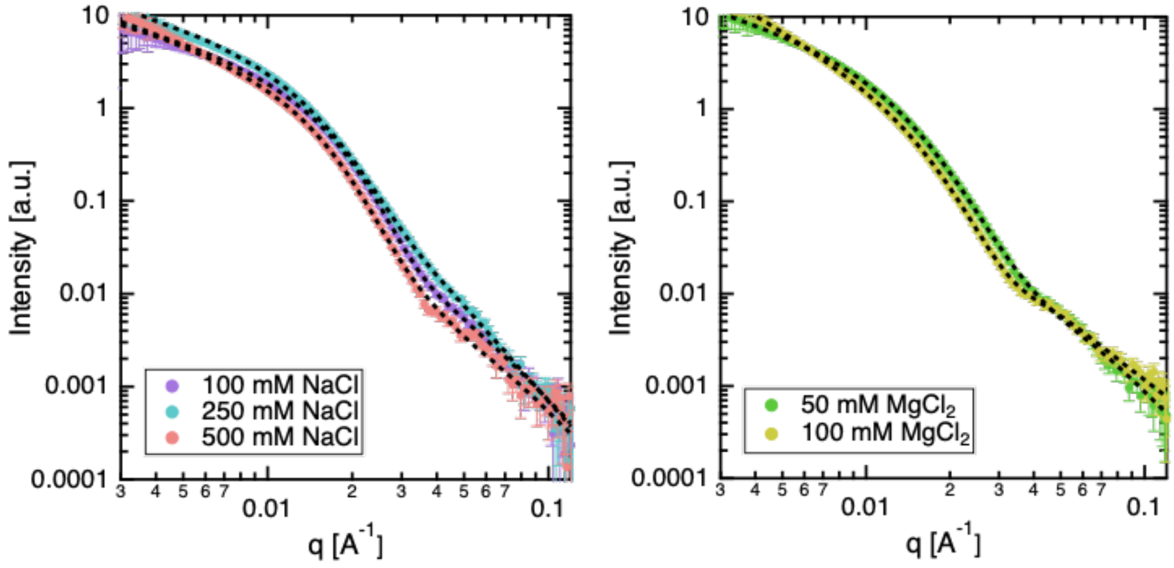

Figure S46. SAXS profiles for PEG_{10K}-PLK_{93} / PAA_{50} with 100, 250 and 500-mM NaCl (left), as well as 50 and 100-mM $MgCl_2$ (right).

S4. Supplemental Polyelectrolyte Complex Micelle Stability Data

Kinetic stability tests of the micelles were examined in FBS. Figure S46 shows the apparent size distribution of PEG-PVBTMA and PMPC-PVBTMA at 1 and 10 h, repeated three times independently. The overall size distribution of particles appears to be consistent, with evidence of PMPC-PVBTMA micelle aggregation over time.

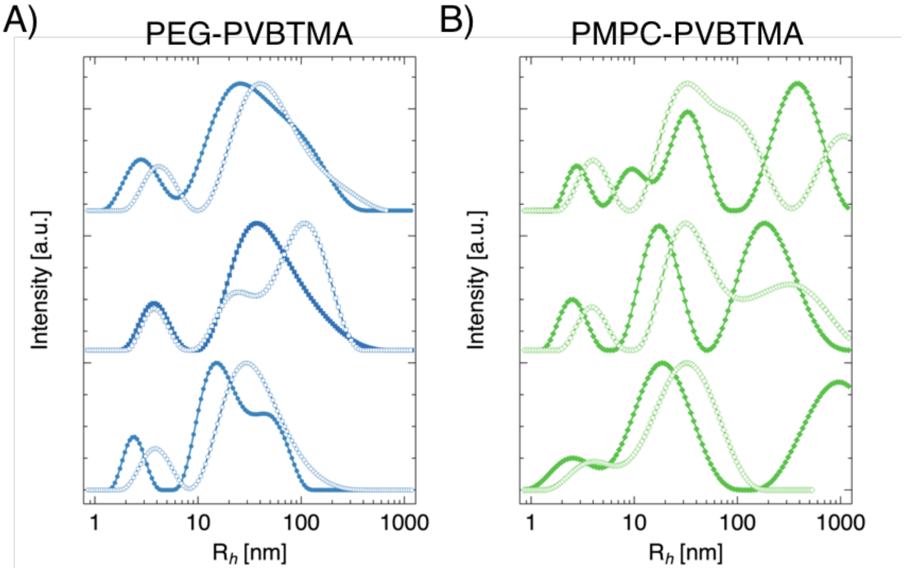

Figure S47. Three replicates of the apparent size hydrodynamic radius distribution of PEG- PVBTMA and PMPC-PVBTMA micelles at 1 h (solid markers) and 10 h (open markers), analyzed by REPES analysis at the 90° angle. Intensity is scaled for clarity.
