# Supplementary material for: Comparing Zwitterionic and PEG Exteriors of Polyelectrolyte Complex Micelles
Source: Molecules. 2020 May 30;25(11):2553. doi: 10.3390/molecules25112553 (PMC7321349; doi:10.3390/molecules25112553)
Supplement: Supplementary file 1 [file molecules-25-02553-s001.zip › molecules-812712-supplementary-revised.docx]

**Supporting Information**

Comparing Zwitterionic and PEG Exteriors of Polyelectrolyte Complex Micelles

Jeffrey M. Ting 1,2,†, Alexander E. Marras 1,2,†, Joseph D. Mitchell 1, Trinity R. Campagna 1 and Matthew V. Tirrell 1,2,*

1 Pritzker School of Molecular Engineering, University of Chicago, Chicago, IL 60637, USA, jting1@uchicago.edu (J.M.T.); marras@uchicago.edu (A.E.M.); jdm41297@gmail.com (J.D.M.); trinityc@uchicago.edu (T.R.C.); mtirrell@uchicago.edu (M.V.T.)

2 Center for Molecular Engineering and Materials Science Division, Argonne National Laboratory, Lemont, IL 60439, USA.

† These authors contributed equally to this work.

Contents

S1. Supplemental Polymer Synthesis Data (Figures S1 to S8)

S2. Supplemental Dynamic Light Scattering Data (Figures S9 to S39)

S3. Supplemental Small-Angle X-Ray Scattering Data (Figures S40 to S46, Table S1)

S4. Supplemental Polyelectrolyte Complex Micelle Stability Data (Figure S47)

S1. Supplemental Polymer Synthesis Data

*PMPC-PVBTMA Synthesis*. Figure S-1 shows a representative crude 1H NMR of PMPC, resulting in 81% total monomer conversion after 18 h. Figure S-2 shows a representative crude 1H NMR of PMPC-PVBTMA, resulting in 93% total monomer conversion after 18 h. Figure S-3 shows the purified 1H NMR of PMPC5K-PVBTMA50.


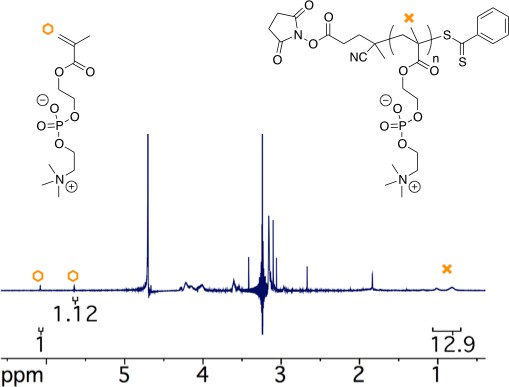


**Figure S1.** Crude 1H NMR of PMPC in D2O.


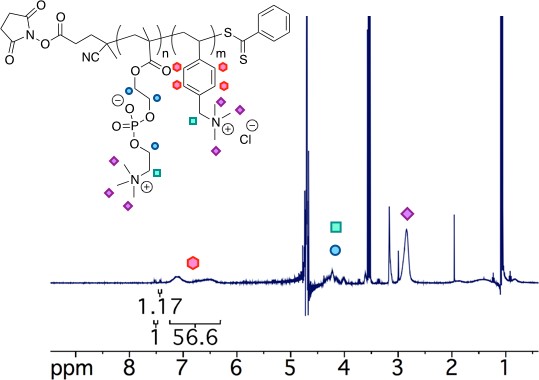


**Figure S2.** Crude 1H NMR of PMPC-PVBTMA in D2O.


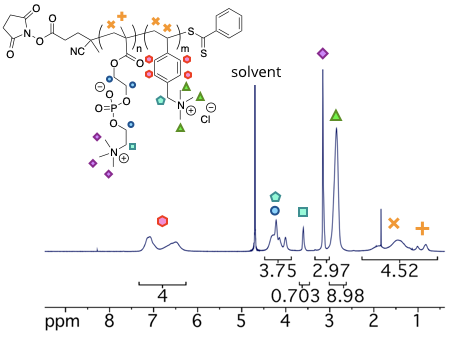


**Figure S3.** 1H NMR of PMPC5K-PVBTMA50 in D2O.

*PAA Synthesis*. Figures S-4 and S-5 show the 1H NMR of the BuPA RAFT CTA and the homopolymer PAA prepared with aqueous RAFT polymerization, respectively. End-group analysis (51 × 94.04 g/mol + 238.39 g/mol) resulted in a calculated *M*n = 5030 g/mol, in excellent agreement with SEC-MALS characterization. Figure S-6 shows the SEC refractive index trace of PAA, which consists of a monomodal peak exhibiting narrow dispersity in the *M*n distribution.


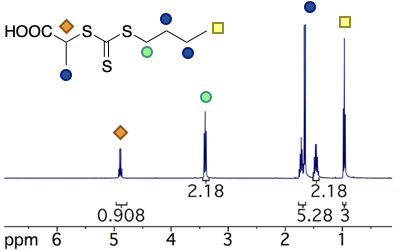


**Figure S4.** 1H NMR of BuPA in CDCl3.


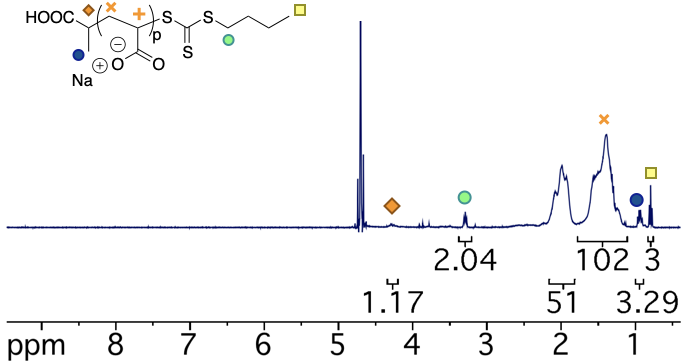


**Figure S5.** 1H NMR of PAA in D2O.


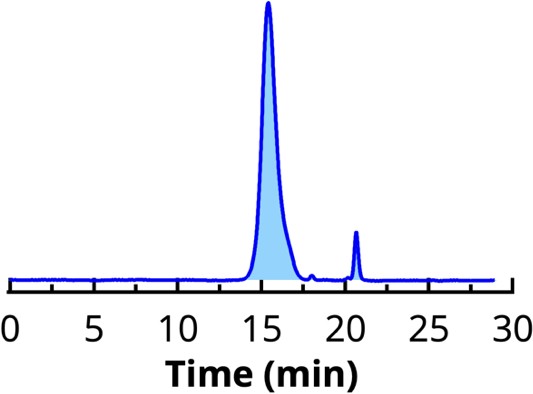


**Figure S6.** SEC refractive index trace of PAA (*M*n = 4900 g/mol, *Đ* = 1.11).

*Refractive Index Measurements*. To determine the absolute *M*n of the polymers measured by SEC-MALS, we employed a refractometer to measure the d*n*/dc of polymers in their respective mobile phase. For PMPC-PVBTMA samples, Figure S-7 shows the d*n*/dc determination of the individual homopolymers. Using Equation 1 shown in the main manuscript, we calculated the d*n*/dc of the PMPC5K-PVBTMA50 and PMPC10K-PVBTMA100 to be 0.1661 and 0.1663 mL/g, respectively.


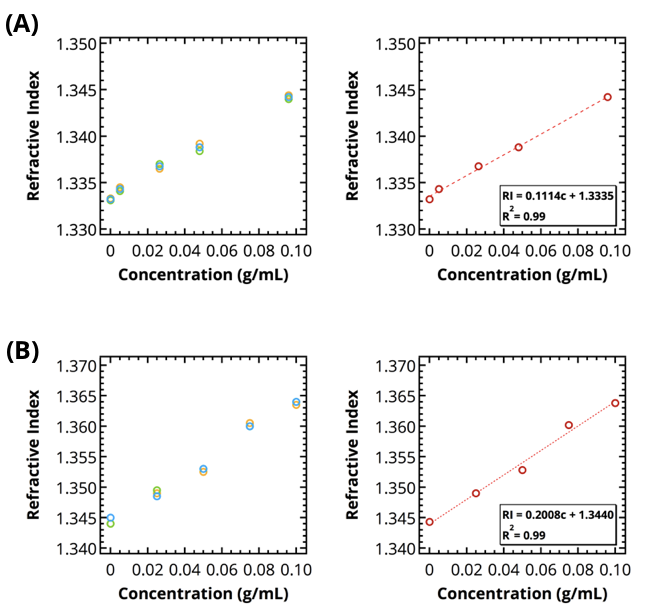


**Figure S7.** Measured refractive index values versus polymer concentration of (A) PMPC and (B) PVBTMA. All measurements were taken using polymers completely dissolved in the cationic mobile phase solution at 25 °C. The circles on the left plot show the raw data; the circles on the right plot show the average of triplicate measurements with the dashed line denoting a linear regression to determine d*n*/dc.

*Thermogravimetric Analysis Curves*. Figure S-8 shows the TGA profiles of synthesized polyelectrolytes. All experiments were conducted at 15 °C/min.


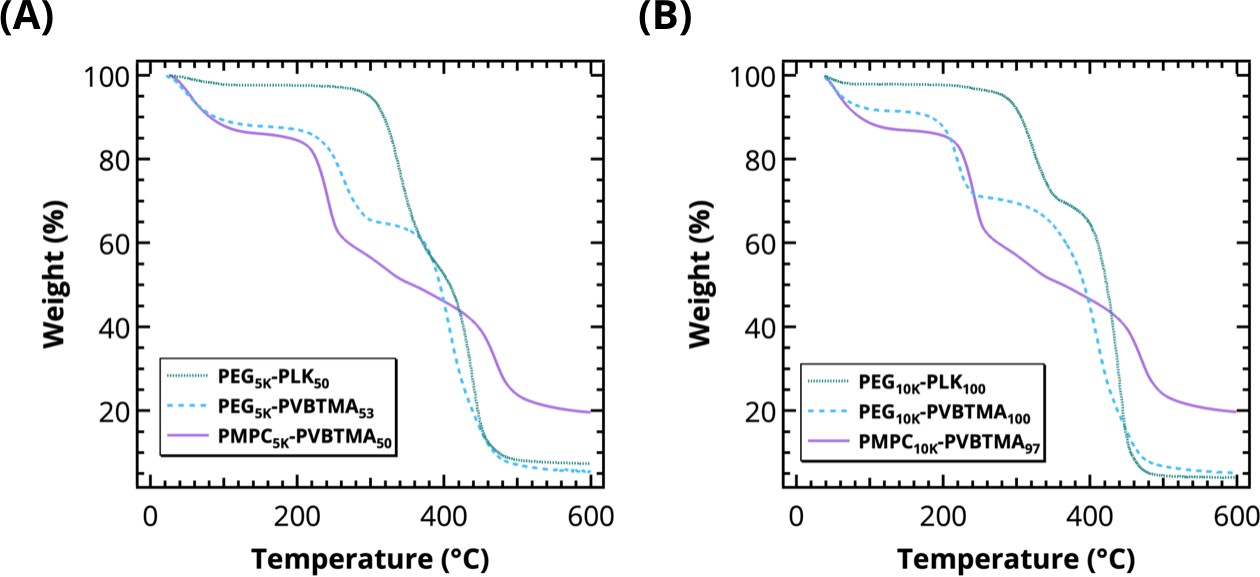


**Figure S8.** TGA curves for block polymers (A) PEG5K-PLK50, PEG5K-PVBTMA53 and PMPC5K- PVBTMA50, as well as (B) PEG10K-PLK100, PEG10K-PVBTMA100 and PMPC10K-PVBTMA97. All experiments were conducted at 15 °C/min.

S2. Supplemental Dynamic Light Scattering Data

Figure S-9 shows a gallery of histograms for all prepared PCMs from 0-mM NaCl to 200-mM NaCl. The autocorrelation functions for these samples are shown in Figures S35 through S38 below.


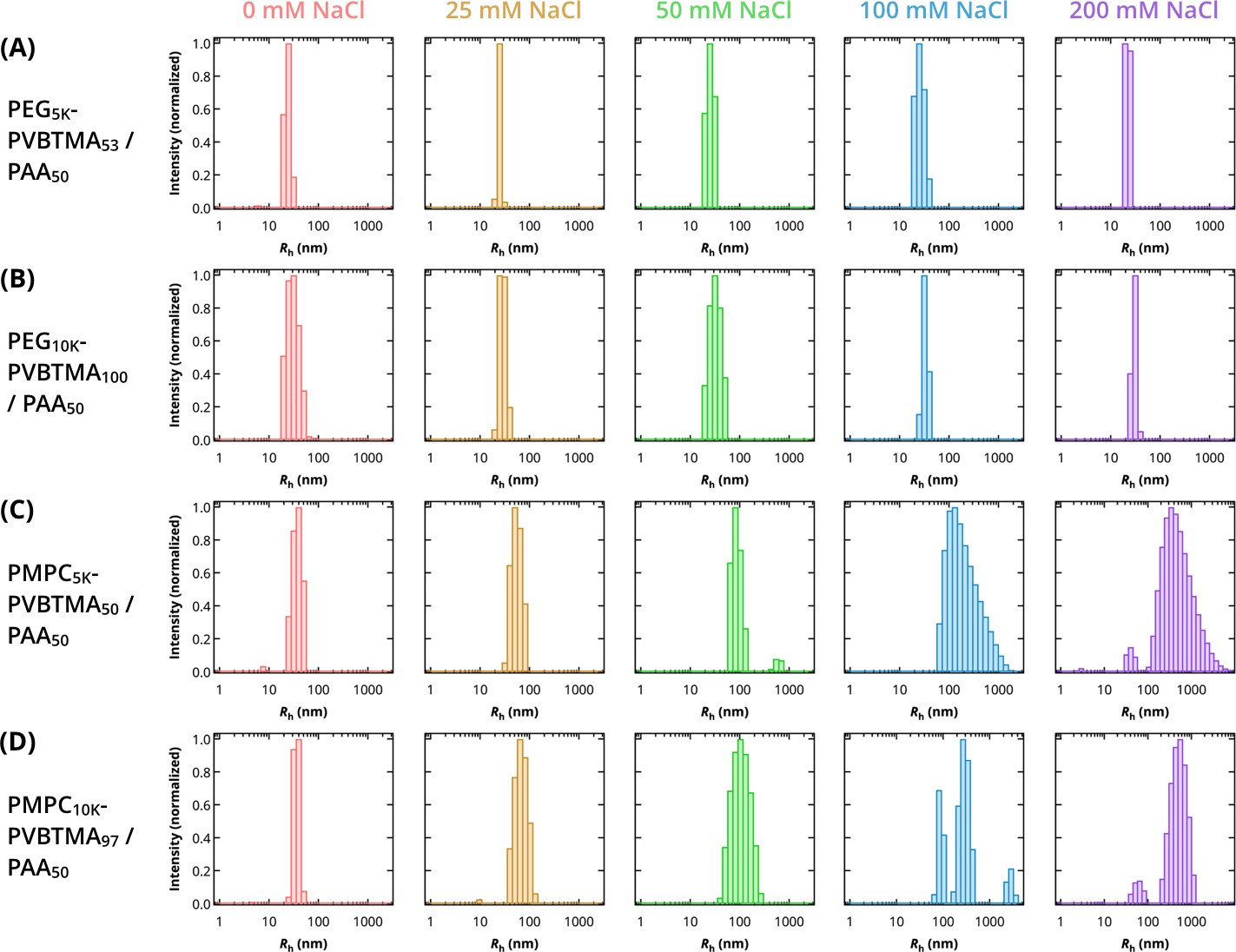


**Figure S9.** Apparent size hydrodynamic radius distribution of (**A**) PEG5K-PVBTMA53 / PAA50,(**B**) PEG10K-PVBTMA100 / PAA50, (**C**) PMPC5K-PVBTMA50 / PAA50 and (**D**) PMPC10K- PVBTMA97 / PAA50 as NaCl salt is increased from 0 mM to 200-mM (left to right).

The detailed multi-angle DLS analysis of the PCMs is provided below. For each polymer system, Figures S10 through S33 show the measured angular dependence of the autocorrelation functions between 60° and 120° fitted by a cumulant expansion, as well as the linear regression of Γ vs *q*2 between 60° and 120°.


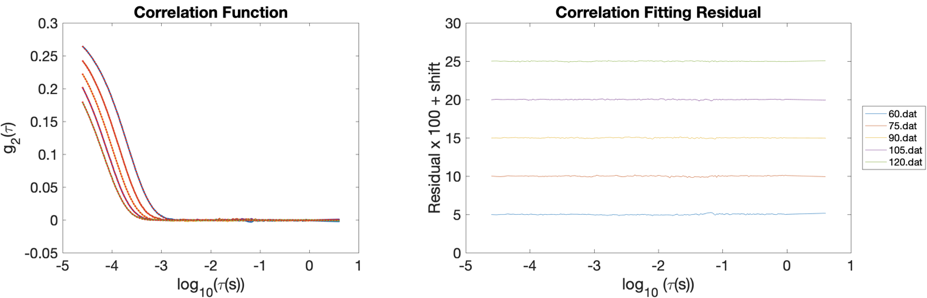


**Figure S10.** Measured angular dependence of the autocorrelation function for PEG5K-PLK47 / PAA50 at 0-mM NaCl between 60° and 120° fitted by cumulant expansion.


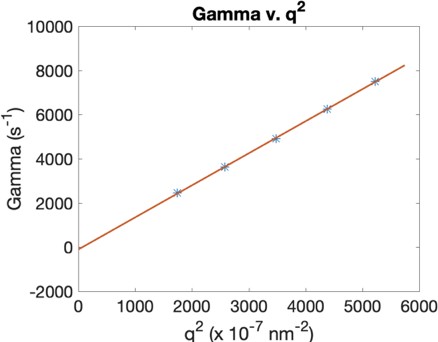


**Figure S11.** Linear regression of Γ vs *q*2 for PEG5K-PLK47 / PAA50 at 0-mM NaCl over 5 angles between 60° and 120°.


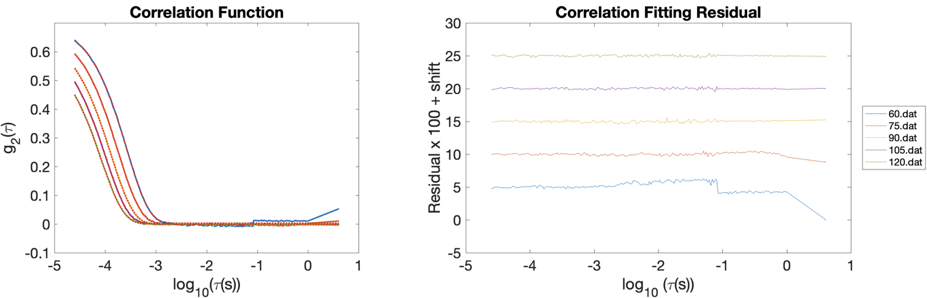


**Figure S12.** Measured angular dependence of the autocorrelation function for PEG5K-PLK47 / PAA50 at 100-mM NaCl between 60° and 120° fitted by cumulant expansion.


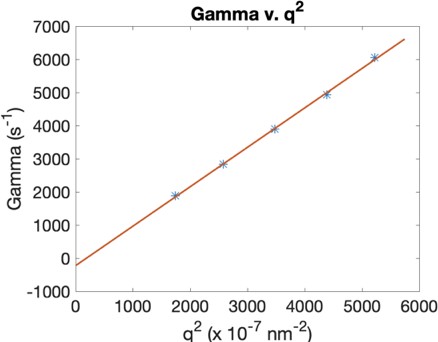


**Figure S13.** Linear regression of Γ vs *q*2 for PEG5K-PLK47 / PAA50 at 100-mM NaCl over 5 angles between 60° and 120°.


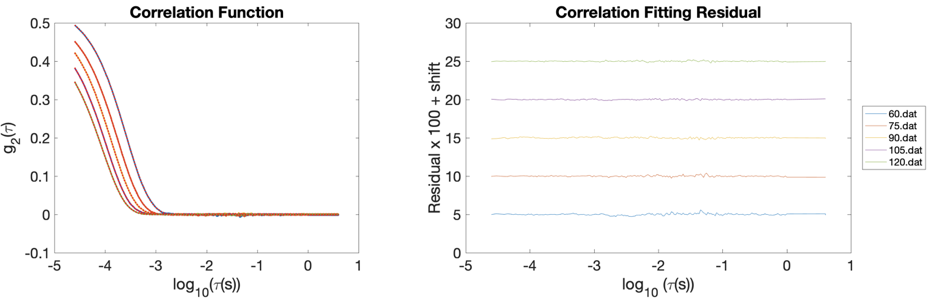


**Figure S14.** Measured angular dependence of the autocorrelation function for PEG10K-PLK93 / PAA50 at 0-mM NaCl between 60° and 120° fitted by cumulant expansion.


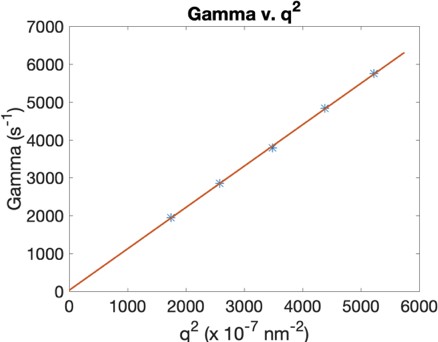


**Figure S15.** Linear regression of Γ vs *q*2 for PEG10K-PLK93 / PAA50 at 0-mM NaCl over 5 angles between 60° and 120°.


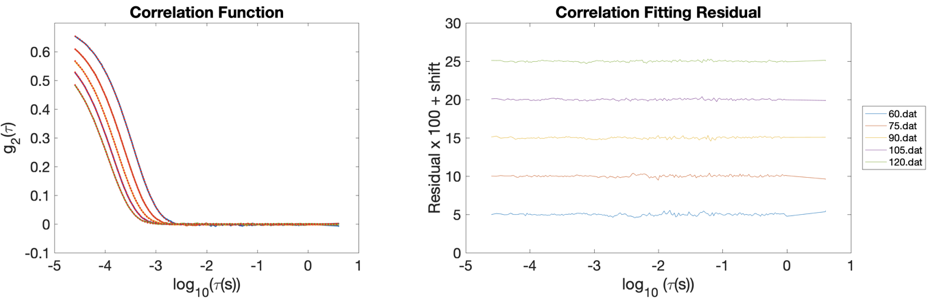


**Figure S16.** Measured angular dependence of the autocorrelation function for PEG10K-PLK93 / PAA50 at 100-mM NaCl between 60° and 120° fitted by cumulant expansion.


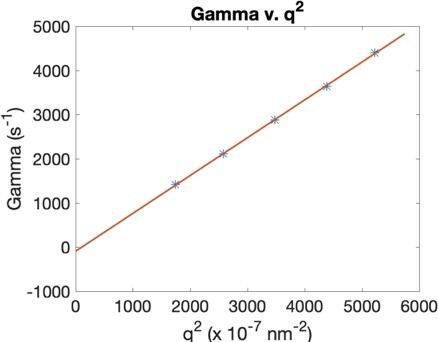


**Figure S17.** Linear regression of Γ vs *q*2 for PEG10K-PLK93 / PAA50 at 100-mM NaCl over 5 angles between 60° and 120°.


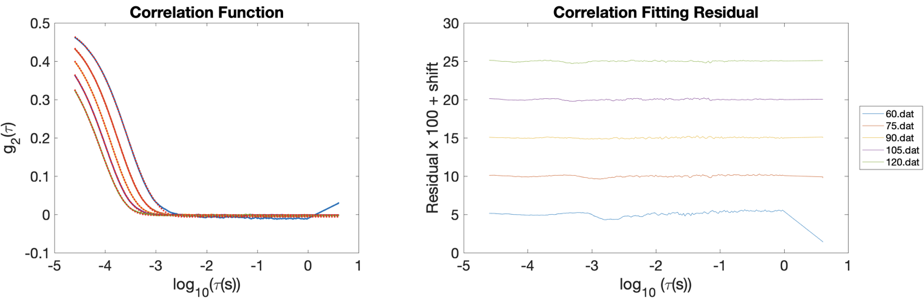


**Figure S18.** Measured angular dependence of the autocorrelation function for PEG5K-PVBTMA53./ PAA50 at 0-mM NaCl between 60° and 120° fitted by cumulant expansion.


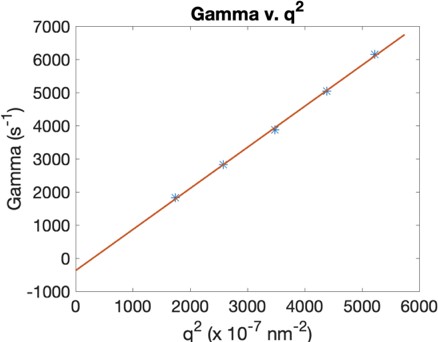


**Figure S19.** Linear regression of Γ vs *q*2 for PEG5K-PVBTMA53 / PAA50 at 0-mM NaCl over 5 angles between 60° and 120°.


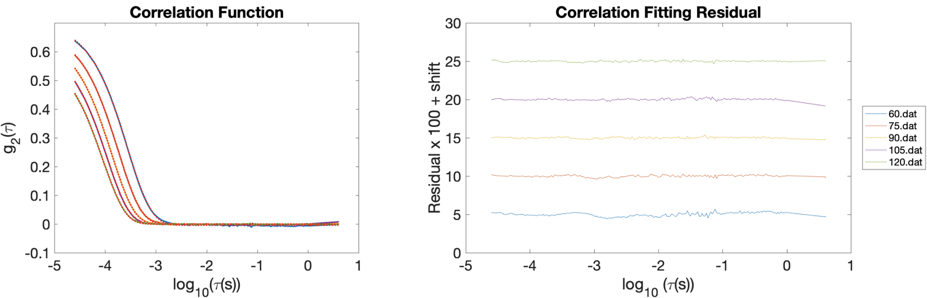


**Figure S20.** Measured angular dependence of the autocorrelation function for PEG5K-PVBTMA53/ PAA50 at 100-mM NaCl between 60° and 120° fitted by cumulant expansion.


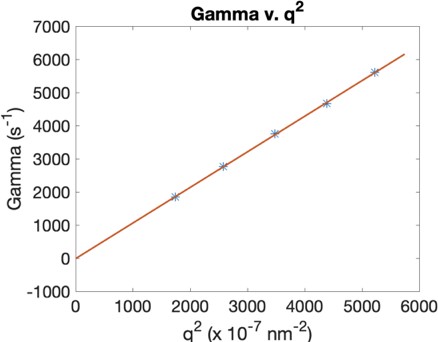


**Figure S21.** Linear regression of Γ vs *q*2 for PEG5K-PVBTMA53 at 100-mM NaCl over 5 angles between 60° and 120°.


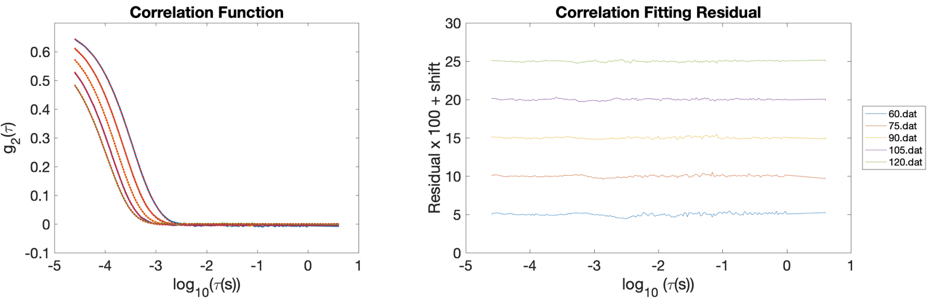


**Figure S22.** Measured angular dependence of the autocorrelation function for PEG10K- PVBTMA100 / PAA50 at 0-mM NaCl between 60° and 120° fitted by cumulant expansion.


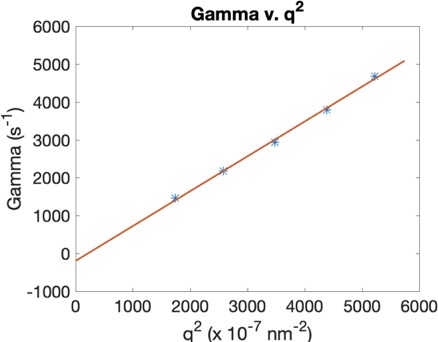


**Figure S23.** Linear regression of Γ vs *q*2 for PEG10K-PVBTMA100 / PAA50 at 0-mM NaCl over 5 angles between 60° and 120°.


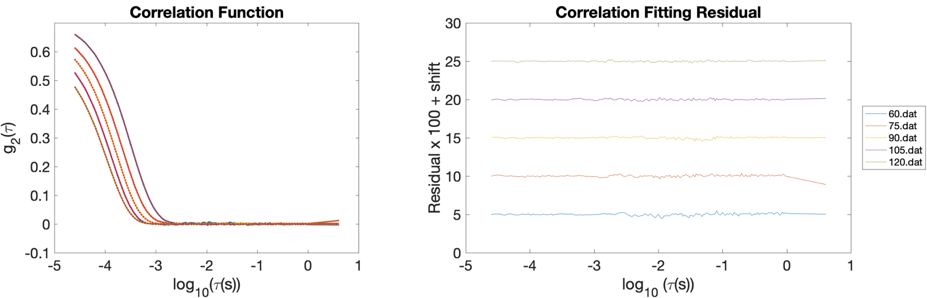


**Figure S24.** Measured angular dependence of the autocorrelation function for PEG10K- PVBTMA100 / PAA50 at 100-mM NaCl between 60° and 120° fitted by cumulant expansion.


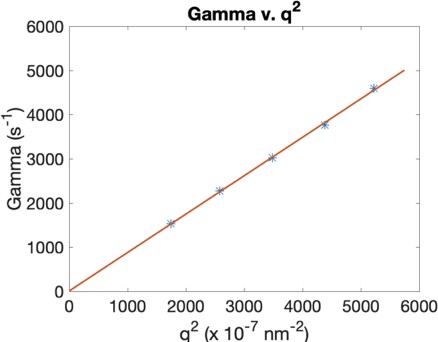


**Figure S25.** Linear regression of Γ vs *q*2 for PEG10K-PVBTMA100 / PAA50 at 100-mM NaCl over 5 angles between 60° and 120°.


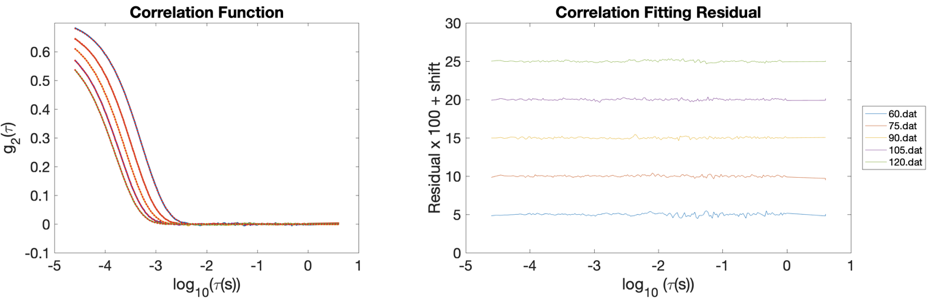


**Figure S26.** Measured angular dependence of the autocorrelation function for PMPC5K-PVBTMA50 / PAA50 at 0-mM NaCl between 60° and 120° fitted by cumulant expansion.


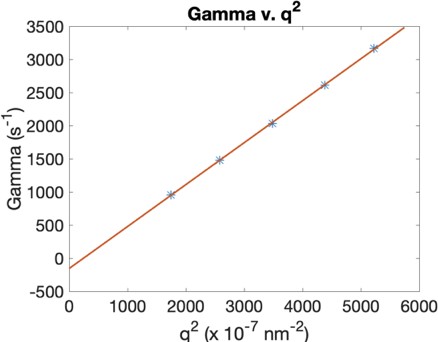


**Figure S27.** Linear regression of Γ vs *q*2 for PMPC5K-PVBTMA50 / PAA50 at 0-mM NaCl over 5 angles between 60° and 120°.


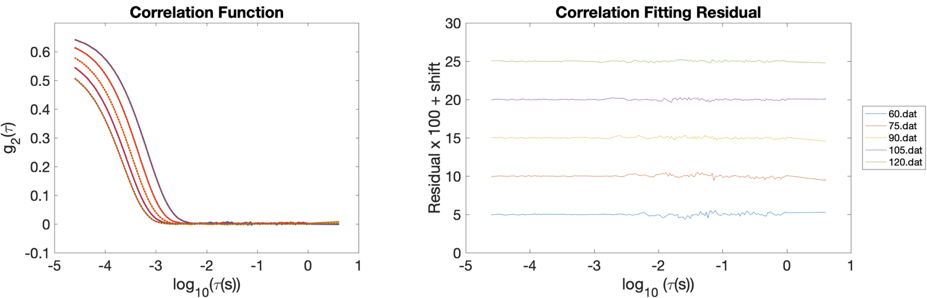


**Figure S28.** Measured angular dependence of the autocorrelation function for PMPC5K-PVBTMA50 / PAA50 at 100-mM NaCl between 60° and 120° fitted by cumulant expansion.


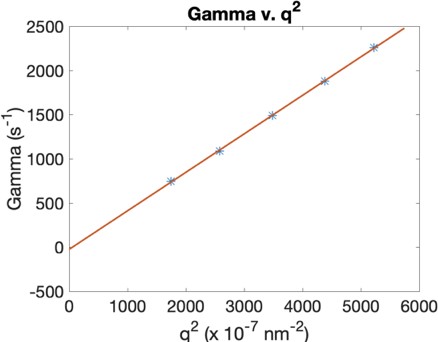


**Figure S29.** Linear regression of Γ vs *q*2 for PMPC5K-PVBTMA50 / PAA50 at 100-mM NaCl over 5 angles between 60° and 120°.


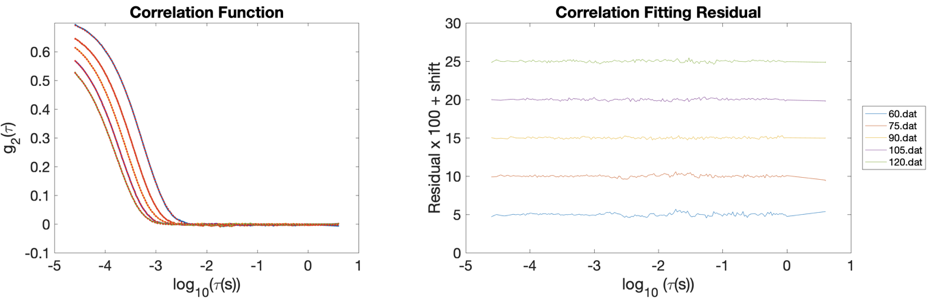


**Figure S30.** Measured angular dependence of the autocorrelation function for PMPC10K- PVBTMA97 / PAA50 at 0-mM NaCl between 60° and 120° fitted by cumulant expansion.


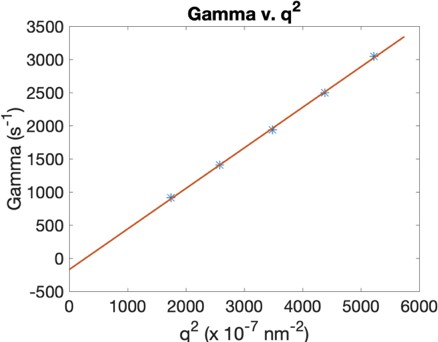


**Figure S31.** Linear regression of Γ vs *q*2 for PMPC10K-PVBTMA97 / PAA50 at 0-mM NaCl over 5 angles between 60° and 120°.


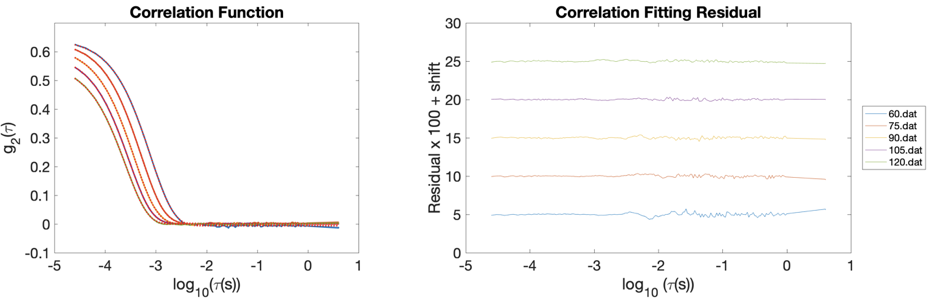


**Figure S32.** Measured angular dependence of the autocorrelation function for PMPC10K-PVBTMA97 / PAA50 at 100-mM NaCl between 60° and 120° fitted by cumulant expansion.


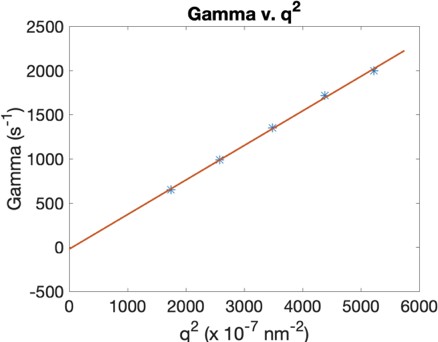


**Figure S33.** Linear regression of Γ vs *q*2 for PMPC10K-PVBTMA97 / PAA50 at 100-mM NaCl over 5 angles between 60° and 120°.

Figures S-34 through S-37 show the autocorrelation functions that correspond to Figure S-9 for all investigated micelle systems from 0 to 200-mM NaCl.


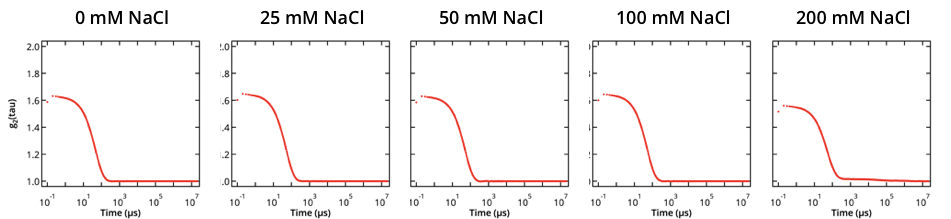


**Figure 34.** Summary of the autocorrelation functions of PCM assemblies PEG5K-PVBTMA53 at 0-200 mM NaCl.


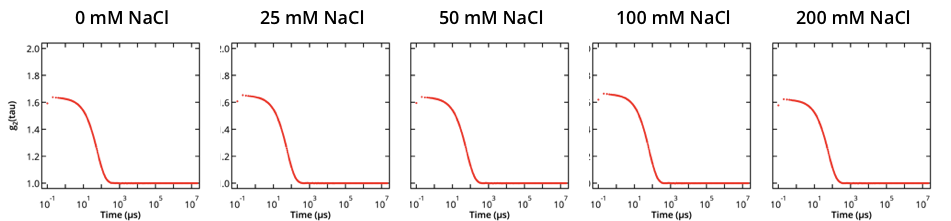


**Figure S35.** Summary of the autocorrelation functions of PCM assemblies PEG10K-PVBTMA100 at 0-200 mM NaCl.


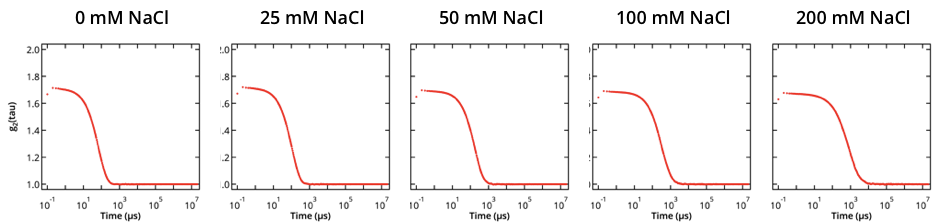


**Figure S36.** Summary of the autocorrelation functions of PCM assemblies PMPC5K-PVBTMA50 at 0-200 mM NaCl.


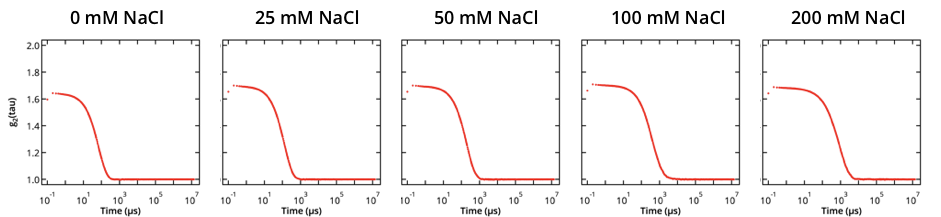


**Figure S37.** Summary of the autocorrelation functions of PCM assemblies PMPC10K-PVBTMA97 at 0-200 mM NaCl.


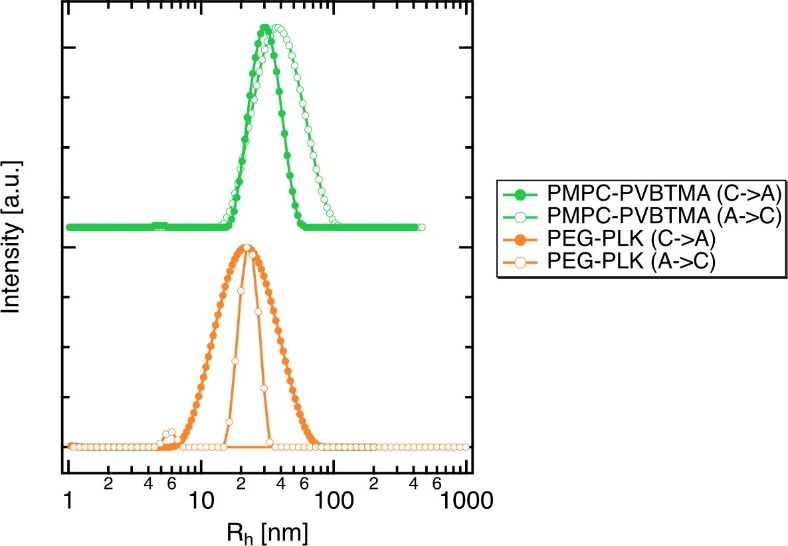


**Figure S38.** REPES fits for DLS of PCMs assembled in different orders. All micelles in this work were assembled with the cation added to the solution first, followed by the anion (CA). When this order is reversed (AC) the size distribution is different, suggesting that the system may be kinetically trapped.


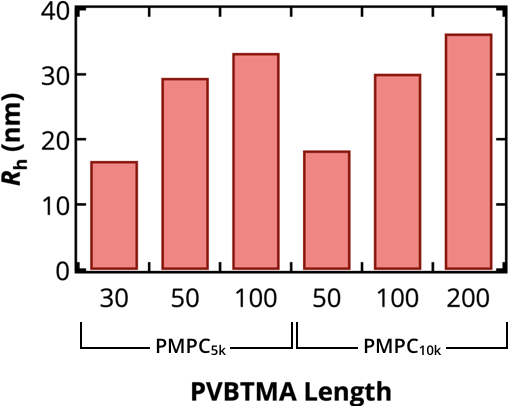


**Figure S39.** Comparison of the apparent hydrodynamic radius versus charged block length of PVBTMA in PCM assemblies.

S3. Supplemental Small-Angle X-ray Scattering

Figure S40 shows the intensity versus q profiles of the PEG-PLK, PEG-PVBTMA and PMPC-PVBTMA polyelectrolyte solutions with increasing NaCl salt. At 0-mM NaCl, the correlation peak can be observed at q = 0.4–0.5 Å for PEG-PLK and q = 0.2–0.4 Å for PEG-PVBTMA and PMPC-PVBTMA. Table S-2 contains the fitting for the SAXS data in Figure 7 of the main manuscript with added MgCl2 salt.


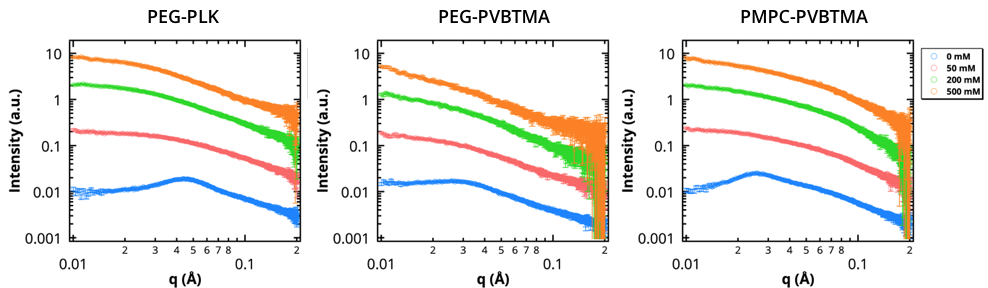


**Figure S40.** SAXS profiles of polyelectrolyte solutions of PEG-PLK, PEG-PVBTMA and PMPC-PVBTMA at polymer concentrations of 5 mg/mL with increasing added NaCl salt. Intensity scaled for clarity.

**Table S1.** SAXS summary with added MgCl2 salt.

| **Sample** | **[MgCl2] (mM)** | ***R*Guinier a (nm)** | **PDI b** |
| --- | --- | --- | --- |
| PEG5K-PLK47 / PAA50 | 50 | 8.0 | 0.16 |
|  | 100 | 8.6 | 0.14 |
| PEG10K-PLK93 / PAA50 | 50 | 11.2 | 0.15 |
|  | 100 | 10.6 | 0.09 |
| PEG5K-PVBTMA53 / PAA50 | 50 | 10.7 | 0.09 |
|  | 100 | - | - |
| PEG10K-PVBTMA100 / PAA50 | 50 | 19.8 | 0.15 |
|  | 100 | - | - |
| PMPC5K-PVBTMA50 / PAA50 | 50 | - | - |
|  | 100 | - | - |
| PMPC10K-PVBTMA97 / PAA50 | 50 | - | - |
|  | 100 | - | - |

a Mean radius from Guinier fit; predominately core (nm) b Polydispersity index (σ2/R2). Models use Schulz-Zimm distribution flexible cylinder and Unified Level fits, except for PEG10K-PVBTMA47 / PAA100 (spheroid) and PEG10K-PLK93 / PAA50 (cylinder fitting).

For each polymer system, Figures S-41 through S-46 show the small angle X-ray scattering profile in water with 100, 250 and 500 mM NaCl as well as 50 and 100 mM MgCl2.


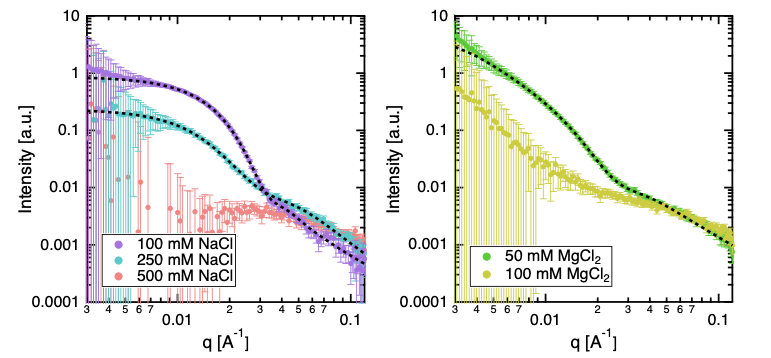


**Figure S41.** SAXS profiles for PEG5K-PVBTMA53 / PAA50 with 100, 250 and 500-mM NaCl (left), as well as 50 and 100-mM MgCl2 (right).


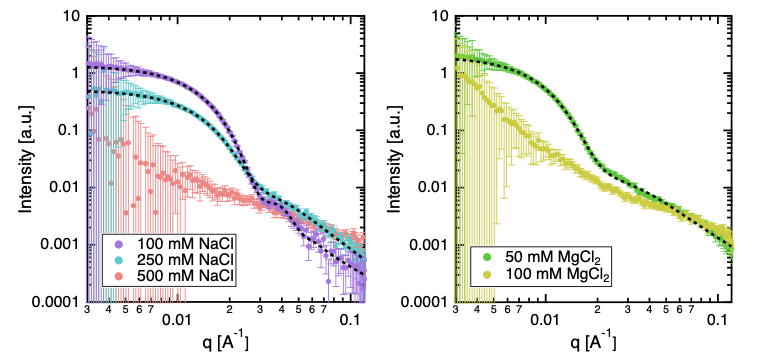


**Figure S42.** SAXS profiles for PEG10K-PVBTMA100 / PAA50 with 100, 250 and 500-mM NaCl (left), as well as 50 and 100-mM MgCl2 (right).


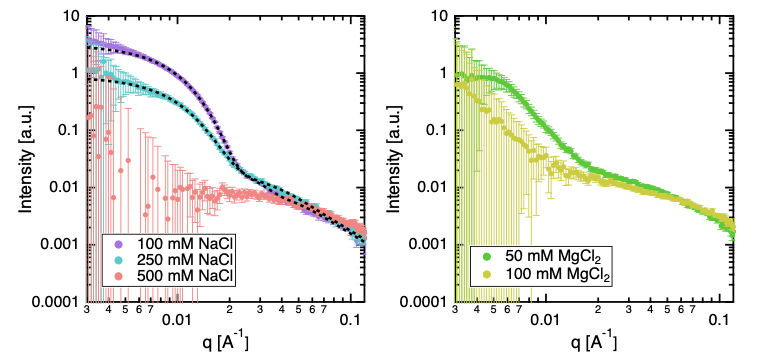


**Figure S43.** SAXS profiles for PMPC5K-PVBTMA50 / PAA50 with 100, 250 and 500-mM NaCl (left), as well as 50 and 100-mM MgCl2 (right).


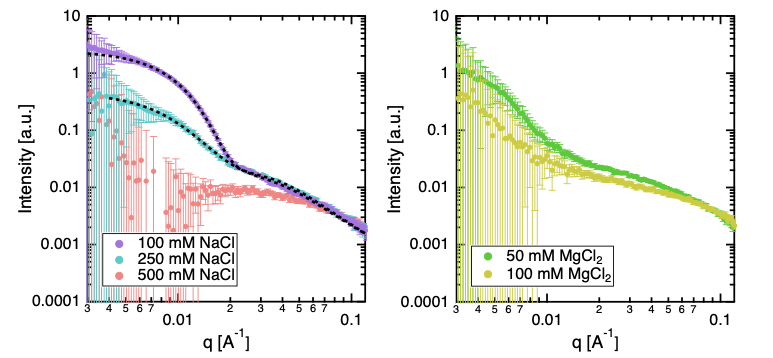


**Figure S44.** SAXS profiles for PMPC10K-PVBTMA97 / PAA50 with 100, 250 and 500-mM NaCl (left), as well as 50 and 100-mM MgCl2 (right).


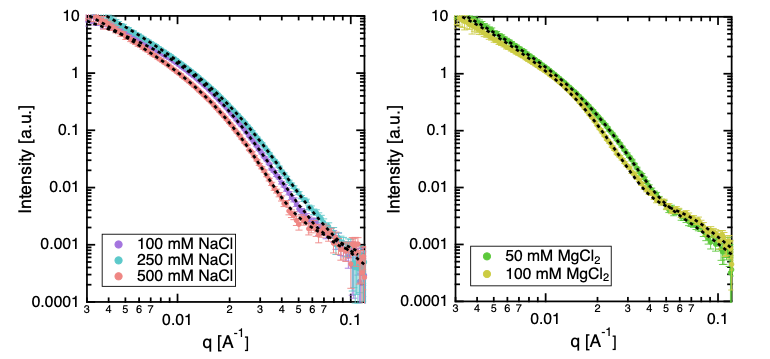


**Figure S45.** SAXS profiles for PEG5K-PLK47 / PAA50 with 100, 250 and 500-mM NaCl (left), as well as 50 and 100-mM MgCl2 (right).


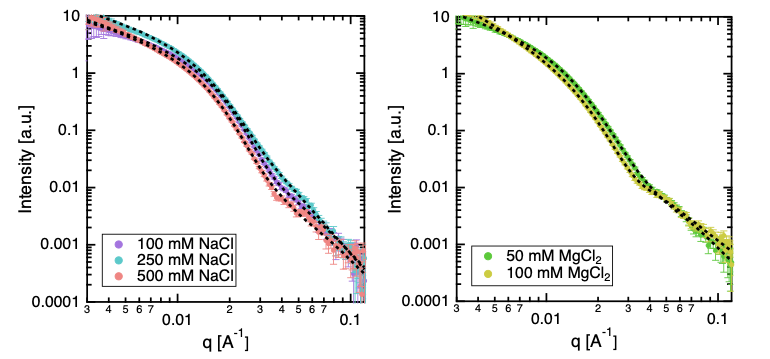


**Figure S46.** SAXS profiles for PEG10K-PLK93 / PAA50 with 100, 250 and 500-mM NaCl (left), as well as 50 and 100-mM MgCl2 (right).

S4. Supplemental Polyelectrolyte Complex Micelle Stability Data

Kinetic stability tests of the micelles were examined in FBS. Figure S46 shows the apparent size distribution of PEG-PVBTMA and PMPC-PVBTMA at 1 and 10 h, repeated three times independently. The overall size distribution of particles appears to be consistent, with evidence of PMPC-PVBTMA micelle aggregation over time.


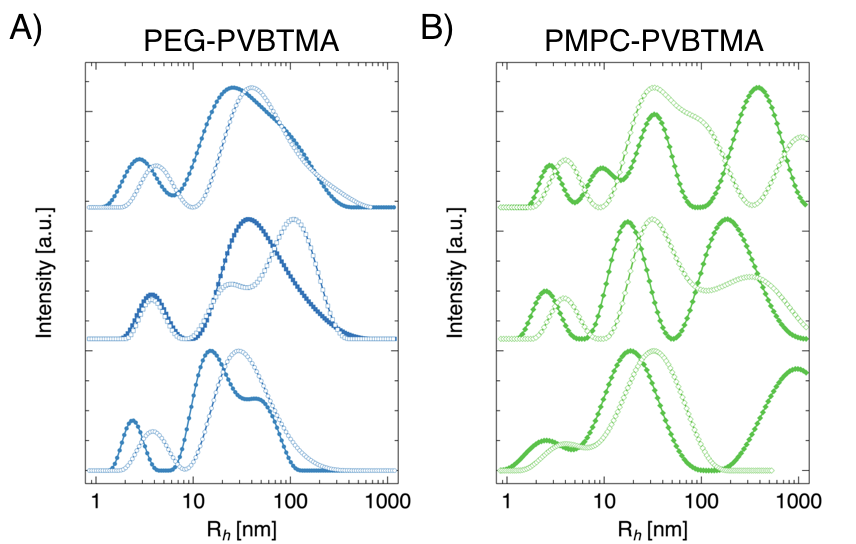


**Figure S47.** Three replicates of the apparent size hydrodynamic radius distribution of PEG- PVBTMA and PMPC-PVBTMA micelles at 1 h (solid markers) and 10 h (open markers), analyzed by REPES analysis at the 90° angle. Intensity is scaled for clarity.
